# Mechanical Behavior of Titanium Based Metal Matrix Composites Reinforced with TiC or TiB Particles under Quasi-Static and High Strain-Rate Compression

**DOI:** 10.3390/ma14226837

**Published:** 2021-11-12

**Authors:** Pavlo E. Markovsky, Jacek Janiszewski, Oleksandr O. Stasyuk, Vadim I. Bondarchuk, Dmytro G. Savvakin, Kamil Cieplak, Daniel Goran, Purvesh Soni, Sergey V. Prikhodko

**Affiliations:** 1 G.V. Kurdyumov Institute for Metal Physics of N.A.S. of Ukraine, 03412 Kyiv, Ukraine; pmark@imp.kiev.ua (P.E.M.); stasiuk@imp.kiev.ua (O.O.S.); vbondar77@gmail.com (V.I.B.); savva@imp.kiev.ua (D.G.S.); 2 Jarosław Dąbrowski Military University of Technology, 00908 Warsaw, Poland; jacek.janiszewski@wat.edu.pl (J.J.); kamil.cieplak@wat.edu.pl (K.C.); 3 Bruker Nano Analytics, Am Studio 2D, 12489 Berlin, Germany; Daniel.Goran@bruker.com (D.G.); Purvesh.Soni@bruker.com (P.S.); 4 Department of Materials Science and Engineering, University of California, Los Angeles, CA 90095, USA

**Keywords:** titanium matrix composite, titanium carbide, titanium boride, microstructure, mechanical properties, high-strain-rate testing, split Hopkinson pressure bar, quasi-static compression, strain energy

## Abstract

The mechanical behavior of titanium alloys has been mostly studied in quasi-static conditions when the strain rate does not exceed 10 s^−1^, while the studies performed in dynamic settings specifically for Ti-based composites are limited. Such data are critical to prevent the “strength margin” approach, which is used to assure the part performance under dynamic conditions in the absence of relevant data. The purpose of this study was to obtain data on the mechanical behavior of Ti-based composites under dynamic condition. The Metal Matrix Composites (MMC) on the base of the alloy Ti-6Al-4V (wt.%) were made using Blended Elemental Powder Metallurgy with different amounts of reinforcing particles: 5, 10, and 20% of TiC or 5, 10% (vol.) of TiB. Composites were studied at high strain rate compression ~1–3·10^3^·s^−1^ using the split Hopkinson pressure bar. Mechanical behavior was analyzed considering strain rate, phase composition, microstructure, and strain energy (SE). It is shown that for the strain rates up to 1920 s^−1^, the strength and SE of MMC with 5% TiC are substantially higher compared to particles free alloy. The particles TiC localize the plastic deformation at the micro level, and fracturing occurs mainly by crushing particles and their aggregates. TiB MMCs have a finer grain structure and different mechanical behavior. MMC with 5 and 10% TiB do not break down at strain rates up to almost 3000 s^−1^; and 10% MMC surpasses other materials in the SE at strain rates exceeding 2200 s^−1^. The deformation mechanism of MMCs was evaluated.

## 1. Introduction

Titanium alloys are an important structural material of modern aerospace, automotive, shipbuilding and military technologies due to the high level of specific strength, fracture toughness, fatigue strength, corrosion resistance, non-magnetization, and some other specific physical–mechanical and service properties [1,2,3,4]. Conventional titanium alloys alloyed with various elements, which are commonly divided into stabilizers of α- or β-phases, do not always meet the requirements for a set of physical and mechanical properties. For instance, these alloys are characterized by rather low wear resistance, fretting corrosion, and relatively low hardness [1,5,6,7]. These drawbacks can be overcome by preparing titanium-based Metal Matrix Composites (MMC) containing some fine hard particles, for example, carbides, borides, etc. [8,9,10]. Another pressing problem is that the choice of materials for new products is usually based on reference data on mechanical properties, which were determined under certain standard, mainly quasi-static, test conditions. In most cases, the standard test settings do not correspond to the actual operating conditions of real equipment exposed to highly dynamic loads. Therefore, to prevent the failure of such structures, designers usually have to apply the so-called “strength margin” approach, which sometimes leads to an increase in the mass of parts and structures at times, and consequently to essential increase in the total weight of products, their price, and operating costs.

The influence of the strain rate on the mechanical behavior of a wide range of titanium alloys has been studied in sufficient details under quasi-static test conditions when the strain rate is somewhere below 10 s^−1^ [11,12,13,14,15,16,17,18], while the number of studies on the same materials in dynamic conditions is more limited [19,20,21,22]. Especially studies focusing on factors such as chemical and phase composition, and in particular, the evolution of the microstructure under high strain-rate conditions, have not been widely deliberated. In addition, such studies are practically absent for MMC based on titanium alloys reinforced with hard particles such as TiC or TiB, which have been shown to be a very promising material for various applications, for example, working under conditions of wear-resistant friction parts, or anti-ballistic protection elements [8,9,10,23,24]. The mechanical behavior of MMC materials at the moment have not been fully investigated with the exception of quasi-static strength conditions. That is why the present study has been devoted to the systematic investigation of the effect of strain rate on mechanical behavior of the widespread two-phase α + β titanium alloy, Ti-6Al-4V (wt.%, Ti64 hereafter), produced using Blended Elemental Powder Metallurgy (BEPM) [25,26,27], as well as MMCs based on this alloy reinforced with particles of TiC or TiB [10]. Dynamic impact compression tests were carried out using a split Hopkinson pressure bar (SHPB) apparatus, and the obtained results were compared with reference data of quasi-static strength experiments.

## 2. Materials and Methods

The alloy Ti64 was fabricated using BEPM; the details of the used technological protocol are described elsewhere [25,26,27]. The starting materials were TiH_2_ powder (particles size < 40 μm), and standard Al-V (60–40 wt.%) master alloy in powder form (particles size < 63 μm). These powders were mixed, cold pressed in the die under a load 250 MPa, and then sintered at 1250 °C for 4 h in a vacuum of 10^−3^ Pa. The resulting material was designated as Ti64BEPM. Samples of MMC based on the same alloy Ti64 reinforced with 5, 10, and 20 (vol.%) of TiC (particles size below 30 μm), or 5, and 10% of TiB (these quantities of hardening particles TiC and TiB were selected based on the results of works [24,28,29]) particles were prepared via the same BEPM technology. The reinforcing particles were introduced to the blend at the mixing stage [10]. Titanium monoboride, TiB, in the final MMC was obtained by adding into a green mixture of titanium diboride, TiB_2_ powder (1÷30 μm). Diboride is chemically converted to monoboride during sintering according to the reaction: Ti + TiB_2_ = 2TiB. The resulting MMCs were designated as Ti64BEPM + XTiC, and Ti64BEPM + XTiB, where “X” denotes the quantity of hardening particles in volume %. All sintered samples were in the form of square bars with dimensions 10 × 10 × 60 mm.

For standard quasi-static tensile (QST) tests, the bars were machined into cylindrical specimens with the gage diameter 4 mm and length 25 mm. Flat samples with dimensions 2 × 9 × 60 mm were prepared for the elastic properties and damping capacity measurements. Finally, cylindrical samples with gauge length and diameter of 5 mm for the dynamic SHPB and quasi-static compression (QSC) tests were cut by the electric discharge machining technique.

Tensile properties were determined following the ASTM E8 standard using the INSTRON 3376 machine. Young’s, Shear moduli, Poisson’s ratio, and Damping capacity of materials were measured with the Resonance-Frequency-Damping Analysis (RFDA) system (IMCE, Genk, Belgium) using Impulse Excitation Technique (IET) following the ASTM E1876-15 protocol. Material microstructures before and after tests as well as the specimens’ fractures were studied using scanning electron microscope (SEM), Vega 3 (Tescan, Czech Republic), equipped with energy dispersive X-ray (EDX) spectroscopy allowing the measurement of the chemical composition of materials. Crystal orientation and phase mapping was performed using Electron Back Scatter Diffraction (EBSD) on bulk samples and Transmission Kikuchi Diffraction (TKD) on electron transparent samples. EBSD and TKD measurements were made using the QUANTAX EBSD system (Bruker, Germany) installed on a Merlin FE-SEM (Zeiss, Germany). Electron transparent samples were prepared from specific locations on the bulk samples using the lift-out method on a Lyra 3 Focused Ion Beam (FIB) SEM (Tescan, Czech Republic). Phase composition of the specimens and their crystalline structure measurements including the texture analysis were performed using X-ray diffraction (XRD) Ultima IV (Rigaku, Japan) system. The gas content within the sintered specimens was measured using a gas analyzer ELTRA OH900.

**Figure 1:**
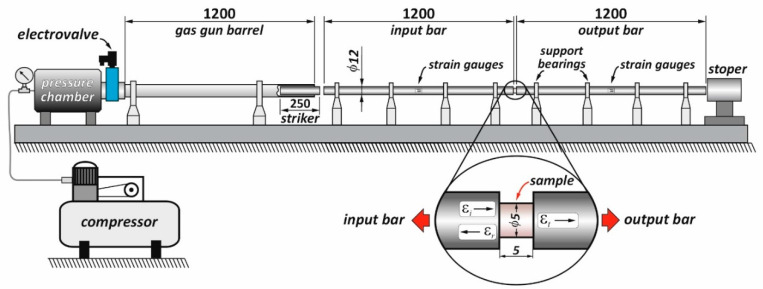
The schematics of the split Hopkinson pressure bar (SHPB) system used in this study [22].

High strain rate compression tests were performed using the SHPB technique [30,31,32]. The basic components of the bar system are shown in Figure 1. The length of the input and output bar was 1200 mm, the length of the striker bar 250 mm and the diameter of all bars 12 mm. The bars were made of heat-treated maraging steel of grade MS350, providing a yield strength of 2300 MPa and an elastic wave speed of 4960 m/s. The striker bar was driven by a compressed air system with a barrel length and inner diameter equal to 1200 mm and 12.1 mm, respectively. The impact striker bar speed used during the experiments ranged from18 to 29 m/s. The pulse shaping technique was used to minimize the wave dispersion and to facilitate stress equilibrium. A copper pulse shaper with a 3-mm diameter and thicknesses of 0.3 or 0.4 mm were used. More details on the applied SHPB stand are given in [22].

## 3. Results

### 3.1. Characteristics of the Original Structures

**Figure 2:**
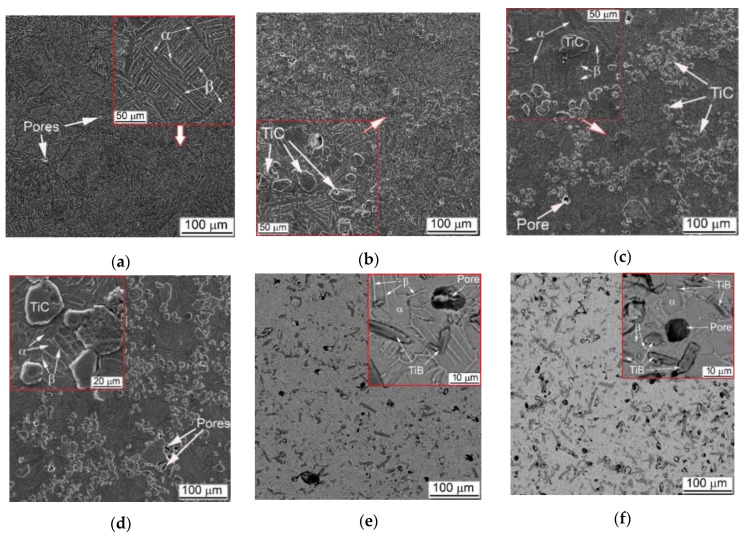
Microstructure of studied materials: (**a**) Ti64BEPM, (**b**–**f**) MMC on the base of Ti64BEPM containing (**b**) 5 (vol.) % TiC, (**c**) 10% TiC, (**d**) 20% TiC, (**e**) 5% TiB, and (**f**) 10% TiB particles. SEM, (**a**–**d**) BSE, (**e**,**f)** SE images.

Typical initial microstructures of the studied materials are presented in Figure 2, and their chemical compositions are listed in Table 1. The measured QST, and 3-point flexure properties, as well as measured elastic characteristics are presented in Table 2. Optimization of sizes of used powders and technological regimes of sintering earlier reported [10] enables to obtain Ti64BEPM alloy homogeneous by chemical composition and having residual porosity below 1.5% (by vol.). As the Figure 2a shows, the prior β-grains’ size was relatively small and equaled 100–150 μm. The intragranular α + β structure was sufficiently fine with the α-lamellas having average length 20 ÷ 70 μm and thickness 2 ÷ 5 μm. The dimensions of all structural elements, such as β-grains, α + β colonies, and individual α-plates (lamellae) are essentially smaller as compared to the same elements of typical coarse-grained lamellar microstructure of Ti64 produced using conventional cast and wrought technology. More details on such comparison were presented earlier [22,24]. The present study structures provide well-balanced standard mechanical properties (tensile) (p. 1 in Table 2), which are superior to those of the lamellar microstructures alloy obtained using conventional cast and wrought method [1,33].

**Figure 3:**
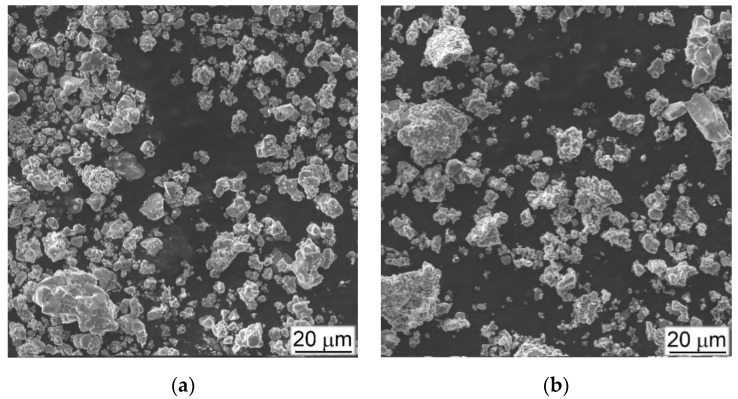
SEM Images (SE) of (**a**) TiC and (**b**) TiB_2_ particles used in preparation of Ti64BEPM + XTiC and Ti64BEPM + XTiB MMCs.

Introduction of reinforcement particles into Ti64BEPM (Figure 3) significantly affects the alloy structure formation during the sintering process [24,34]. TiC particles are considered relatively stable at the used sintering conditions retaining their close to equiaxed morphology (Figure 2b); however, some possibility of partial diffusion of carbon atoms into the matrix alloy solid solution cannot be completely excluded in the light of recent studies [35,36]. It should also be noted that mixing of the alloy blend with TiC particles often accompanied by agglomeration of the particles forming some aggregates and leaving some singular particles. This phenomenon increases with increasing content TiC (Figure 2c,d). The presence of conglomerates and individual TiC particles provides a pinning effect restricting the growth of β-grains in the Ti64 matrix alloy, which do not exceed 50–70 microns in the sintered state of the composite, against 100–150 microns for material without reinforcing particles (Figure 2b–d vs. Figure 2a). As the fraction of TiC particles increases, the residual porosity also increases slightly as shown in Table 1. The structure of composite was somewhat different in the case of TiB reinforcement. These particles are the result of an in-situ chemical reaction during sintering, which transforms more or less equiaxial TiB_2_ particles into needle- or plate-like TiB (Figure 2e,f). As recently shown [37], the TiB particles growth process is accompanied by the Kirkendall effect, which causes a higher total porosity compared to the alloy or its composite with TiC. In addition, the TiB composites had a substantially uniform distribution of reinforcing particles without visible aggregates. All these effects are discussed in more details in previous studies [10,26,27,38].

As previously discussed, [10,26,27] the main reason for the observed microstructure refinement is the presence of residual pores, which pins the β-grain boundaries, thus preventing the grains coarsening during the structure sintering at single β phase field temperatures. A finer β-grain structure in turn affects the size of intra-grain structure that forms in α + β phase field during cooling. The tensile properties of Ti64BEPM alloy compared to the cast and wrought Ti64 with lamellar microstructure [22] were somewhat improved in strength (Table 2, p. 1), most likely due to a higher oxygen content (Table 1, p. 1). In turn, lower ductility can be associated with both: increased oxygen content and residual pores (2% and above). The addition of TiC particles changes the coarseness of both β-grains and intragranular lamellar structures, because these hard particles play the same pinning role as residual pores, and naturally this effect is enhanced as the number of particles increases (compare Figure 2a–d). For instance, in Ti64BEPM + 5TiC alloy in regions relatively free of particles, the grain size is about 100 μm (Figure 2b), while in their presence the grain size varied within the range 10 ÷ 70 μm. The sizes of the colonies and individual lamellas associated with grain size also decreases. A similar trend is generally valid for the higher contents of TiC composites (Figure 2b–d). Except that the clustering of the TiC particles becomes more pronounced at a higher particles content; and it also increases the value of residual porosity from about 1.5% in Ti64BEPM to 2.1%, 2.4%, and 2.8% for Ti64BEPM + 5TiC, Ti64BEPM + 10TiC and Ti64BEPM + 20TiC, respectively (Figure 2a–d). Given the previously established fact that an increase in residual porosity in the range of 1–2% has little effect on the tensile properties of the Ti64 alloy [24,25,26], the almost absolute brittleness of MMC with 5% and above TiC (compare p. 1 with pp. 2–4 in Table 2) should be related to the presence effect of hard particles. In addition to ductility, the strength of the material also decreases with an increase in the number of particles. The UTS is reduced from 1033 MPa for Ti64BEPM to 708 Mpa, 620 Mpa and 567 Mpa for 5%, 10%, and 20% TiC, respectively.

Due to more uniform distribution of TiB particles in the respective composites, the distance between the strengthening particles is shorter than in TiC-containing composites, which causes the β-grains refinement below 50–60 μm. Better refinement of TiB composites causes that in smaller β-grains at 5% TiB the α-phase still has a shape of relatively short lamellae (Figure 2e), while at 10% of TiB particles the α-phase already has a morphology close to globular (Figure 2f). As for the mechanical characteristics of MMCs with TiB under the tension and the 3-point bending test, the strength is markedly lower compared to similar data of TiC composites with alike particles content (Table 2, pp. 5 and 6). This is apparently due to the higher number of residual pores (pp. 5 and 6 in Table 2) and possibly to the effect of the particle morphology, when the sharp edges of TiB plates and needles act as stress concentrators [24].

**Figure 4:**
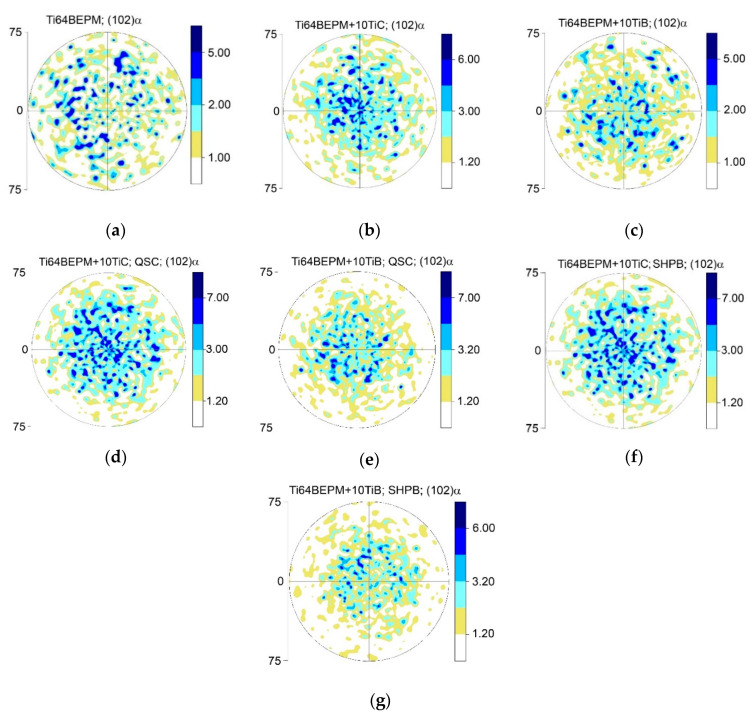
The pole figures (102) α of Ti64BEPM (**a**), Ti64BEPM + 10TiC (**b**,**d**,**f**), and Ti64BEPM + 10TiB (**c**,**e**,**g**); in initial (as-sintered) state (**a**–**c**), and after quasi-static (**d**,**e**), and high-strain rates (**f**) 1550 s^−1^, and (**g**) 2050 s^−1^ compression.

The XRD results confirm that the matrix of alloy is two-phase α + β base with a small amount of β-phase, and the presence of TiC or TiB phases confirmed if reinforcing particles were added to the blend before the sintering. The typical (102)α pole figures of as-sintered specimens are presented in Figure 4a–c. The Ti64BEPM is characterized by a random crystallographic texture, quite typical of a metal having relatively large (~100 μm) prior β-grains (Figure 4a). The size of these grains determines the size of the α-lamellas packets within the grains that gives corresponding reflections on these pole figures. Compared to a reinforcement-free alloy, composites with 10% of hard particles exhibit a higher density of α-phase spots closer the center of the pole figures than on their periphery (Figure 4b,c vs. Figure 4a). A slight decrease in the size of the spots of the (102) α reflection can also be noted, which can be attributed to already mentioned decrease in the average size of β-grains and α-packets caused by presence of particles TiC or TiB (Figure 2c,f vs. Figure 2a).

### 3.2. Compression Tests

**Figure 5:**
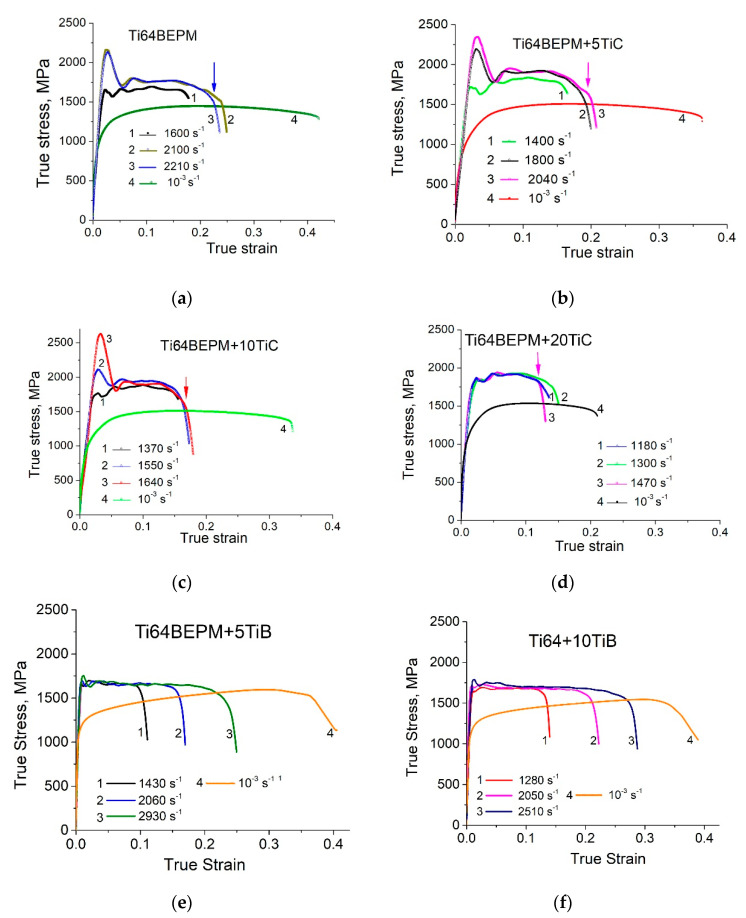
Typical examples of true-stress–true strain curves for SHPB (##1–3), and QSC (# 4) tests obtained with: (**a**) Ti64BEPM, (**b**) Ti64BEPM + 5TiC, (**c**) Ti64BEPM + 10TiC, (**d**) Ti64BEPM + 20TiC, (**e**) Ti64BEPM + 5TiB, and (**f**) Ti64BEPM + 10TiB samples tested at different rates. Arrows (their colors match the colors of the relevant curves) indicate the moments of samples’ cracking.

Typical results of compression test with different strain rates of all studied samples BEPM fabricated are shown in Figure 5. It can be emphasized that the general view of the stress-strain curves of BEPM-made materials tested at high- strain-rate is rather the same as for a conventional cast and wrought Ti64 alloy with lamellar microstructure (Ti64LM) as was described in earlier work [22]. A comparison of the two materials manufactured by different technologies shows that for approximately the same strain rate levels, the maximum strength and strain levels were significantly higher for Ti64BEPM material than for conventional ones Ti64LM. As a result, the fracture of Ti64BEPM samples occurs at higher strain rates of more than 2200 s^−1^, while cast and wrought samples of Ti64LM break at strain rates below 2000 s^−1^ [22]. This behavior was explained by finer microstructure of Ti64BEPM, ensuring its better plasticity. The addition of 5% TiC at first glance causes only minor changes in stress-strain curves compared to non-reinforced with TiC alloy Ti64BEPM (Figure 5b vs. Figure 5a). However, a more detailed analysis of stress-strain curves shows that at similar strain rates the plastic flow stress level increases while the cracking strain values decrease only slightly (compare, for instance curves 1 and 2 in Figure 5a with curves 2 and 3 in Figure 5b). In addition, the introduction of 5% TiC reduces the strain rate at which fracture occurs from 2210 s^−1^ to 2040 s^−1^. An increase in TiC content of up to 10% boosts this trend, leading to an increase in plastic flow stress and a decrease in cracking strain (Figure 5c).

Finally, at 20% of TiC, the plastic flow stress reaches its maximum level of 1800÷1900 MPa, while the cracking strain is noticeably reduced by about half compared to Ti64BEPM + 5TiC. This results in samples breaking at relatively low strain rate of 1470 s^−1^, which is the lowest among all the materials studied in the present and previous [22] works.

The high strain-rate curves of the Ti64BEPM hardened with TiB particles have significant differences compared to the MMCs hardened with TiC. First of all, surprisingly, having slightly lower plastic flow stress level (1600–1700 MPa for the Ti64BEPM + TiB versus 1700–1900 MPa for the Ti64BEPM + TiC), these MMCs are characterized by much higher plasticity, and they were not broken even at the strain rates up to 3000 s^−1^ (Figure 5e,f). Such superiority of TiB-hardened MMCs over their counterparts hardened by TiC seems to be quite unexpected. In earlier discussed QST and 3-point bending tests results (Table 2), the relation between characteristics of strength and ductility was the opposite. However, as will be shown below, the compressive load fundamentally changes the nature of the mechanical behavior of these materials when compared to the tensile load.

QSC tests for all MMCs materials hardened with TiC particles show the similar plastic flow stress level of about 1300–1400 MPa, which is slightly higher than for the base alloy Ti64BEPM (curves#4 in Figure 5a–d). The main difference between these four materials is a gradual decrease in the cracking strain: from 0.43–0.5 for Ti64BEPM to 0.22–0.25 for Ti64BEPM + 20TiC. In turn, the QSC stress-strain curves of MMCs with TiB reveal marked differences from previous cases. First, the achievable strength exceeds 1500 MPa in combination with a strain greater than 0.35 (curves 4 in Figure 5e,f). Second, the Ti64BEPM + TiC showed a small strain hardening effect for strain range to 0.1, while Ti64BEPM + TiB revealed a very small negative strain hardening (strain softening effect). Interestingly, the Ti64BEPM + TiB deformed in quasi-static conditions does not show such behavior, whereas in the case of composite Ti64BEPM + TiC, the strain hardening mechanism has almost the same course, both in quasi-static and dynamic deformation. A positive slope of the Ti64BEPM + TiC curves can be associated with either dynamic matrix strengthening or material compaction due to pore collapsing. In turn, negative slope of the high strain-rate curves for the Ti64BEPM + TiB materials can be resulted in cracking the TiB particles under dynamic deformation. It should also be noted that increasing the TiB content from 5 to 10% practically does not change the mechanical characteristics of the material.

## 4. Discussions

### 4.1. Strain Energy

**Figure 6:**
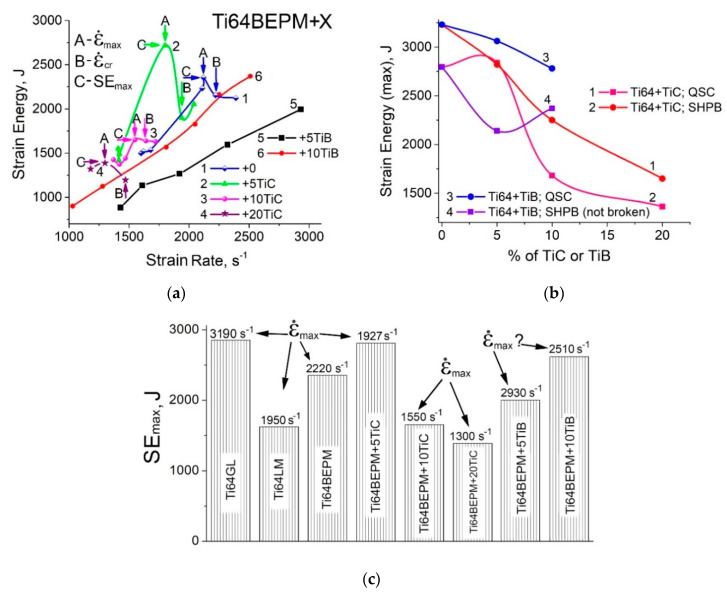
The strain energy *SE*: (**a**) vs. strain rate dependencies for studied Ti64BEPM based materials without (1) and MMCs with (2–4) TiC, and (5,6) TiB particles; (**b**) The strain energy *SE* for QSC with rate 10^−3^ s^−1^ and high-strain-rates SHPB compressions vs. content of TiC or TiB particles; (**c**) the *SE_max_* and $\dot{\varepsilon }$_max_ values for tested materials compared with the data for Ti64GL and Ti64LM taken from [22]. Vertical arrows in (**a**) indicates the strain rates at which specimens fracture. A, B, and C in (**a**) indicate measured values of $\dot{\varepsilon }$ _max_, $\dot{\varepsilon }$ _cr_, and *SE_max_* respectively. The “?” sign in (**c**) above the Ti64BEPM + 10TiB bar means that it is not known whether this value is the limit, since the sample was not broken and it was not tested at a higher strain rate.

In order to carry out more in-depth assessment of the mechanical behavior of the materials tested under dynamic loading, an additional parameter, i.e., elastic–plastic strain energy (*SE*), was used. It is a convenient parameter that allows comparing the mechanical response of materials tested with various methods and strain rates [13,14,15,16,17]. The SE is defined as the internal work performed to deform a material specimen through an action of the externally applied forces. The *SE* was determined by integrating the area under the stress–strain curve. In the case of the cracked specimens, a value of *ε*_upper_ corresponded to a value of strain at fracture, whereas for the non-cracked specimens, *ε*_upper_ was assumed to be equal to strain at the moment of the specimen unloading (sharp drop in stress–strain curve). The upper integration limit *ε*_upper_ for the non-cracked specimens under quasi-static loading was assumed to be 0.5. The *SE* values calculated from the experimentally obtained stress-strain curves and plotted vs. strain rate are shown in Figure 6a. The Ti64BEPM samples demonstrate the highest *SE* values among all previously studied [22] Ti-based materials when tested at strain rates up to 2200 s^−1^ (A and C arrows on the curve #1). At higher strain rates (B arrow, ibid.), the Ti64BEPM samples break and the *SE* values drops substantially.

The addition of a reinforcing phase to the alloy significantly alters the mechanical behavior of the sintered material. For the 5% TiC MMC, the *SE*, including its value at maximum, the *SE_max_*_,_ is essentially higher compared with the Ti64BEPM alloy not hardened by particles. However, the 5%TiC MMC fractured at a critical strain rate, $\dot{\varepsilon }$_max_, 1927 s^−1^, which is significantly lower in comparison to alloy without reinforcement, $\dot{\varepsilon }$_max_ = 2220 s^−1^ (Figure 6a, curves 1 and 2 respectively). A further increase in the TiC content to 10% and 20% decreased both the *SE_max_* and $\dot{\varepsilon }$_max_ values (Figure 6a, curves 3 and 4).

The reinforced TiB MMCs are characterized by lower *SE* values for the same strain rates in comparison with Ti64BEPM containing similar amounts of TiC: curves 5 and 6 vs. curves 2 and 3, correspondingly (Figure 6a). However, the containing TiB MMCs did not fracture in the entire range of tested strain rates, as opposed to titanium carbide reinforced ones. As a result, the lowest *SE* values for 5%TiB at strain rates below 2000 s^−1^ were obtained; however, the *SE* values for 10%TiB MMCs become markedly higher (curves 5 and 6). A further increase in the strain rate of the 10%TiB MMC specimens above about 2250 s^−1^ results in an increasing the *SE* level exceed all other tested materials. If we compare these materials in terms of the *SE_max_*_,_ then the Ti64BEPM + 10TiB MMC has superiority over Ti64BEPM + 10TiC (curves 4 and 2 in Figure 6b). In turn, for the QSC testing condition, the *SE* values for both Ti64BEPM + TiB MMCs are higher than for Ti64BEPM + TiC with the same amount of TiB and TiC used. The difference in the *SE_max_* values increase with increasing content of reinforcing particles (Figure 6b, curve 3 vs. curve 1). It can also be noted that the *SE_max_* values for the SHPB and QSC tests were approximately the same only for Ti64BEPM + 5TiC material (Figure 6b, curves 1 and 2). For all others materials, the *SE_max_* values were much higher for QSC compared to the obtained in the SHPB tests (curves 1 and 3 vs. 2 and 4, ibid.).

The comparative overview of the *SE_max_* values at the corresponding maximum strain rates, $\dot{\varepsilon }$_max_, for different materials on the base of Ti64 alloy studied here and also earlier reported data are shown in Figure 6c. It can be seen that the best combination of parameter *SE_max_* and $\dot{\varepsilon }$_max_ corresponds to cast and wrought Ti64 with an “ideal” globular microstructure (2795 J at 3190 s^−1^), and Ti64BEPM + 5TiC (2840 J at 1927 s^−1^). The next materials in decreasing order of *SE_max_* and $\dot{\varepsilon }$_max_ parameters are Ti64BEPM + 10TiB (2370 J at 2510 s^−1^) and Ti64BEPM + 5TiB (2140 J at 2930 s^−1^), and finally Ti64BEPM + 10TiC and Ti64BEPM + 20TiC, for which the lowest values were obtained as a result of premature fracture of the samples (Figure 6c).

### 4.2. Changes in Phase Composition, Microstructure and Micro-Texture

#### 4.2.1. MMC Reinforced with TiC

**Figure 7:**
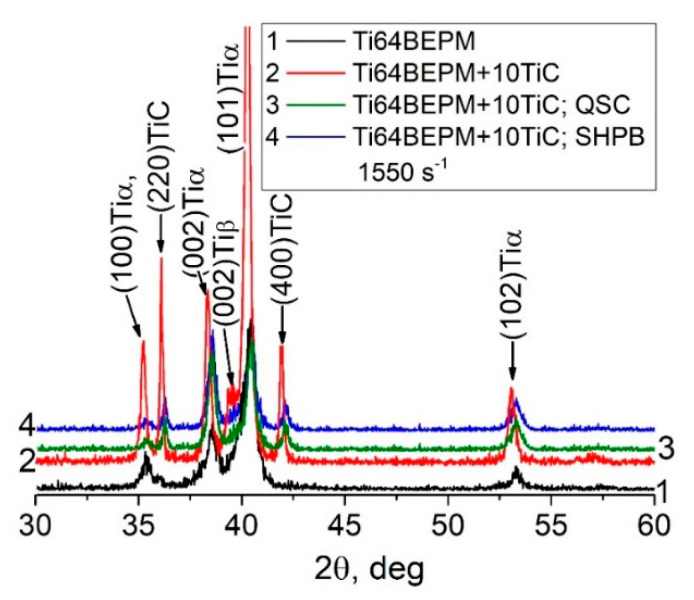
The X-ray diffraction patterns of (curve 1) Ti64BEPM, and Ti64BEPM + 10TiC in (curve 2) as-sintered state, and after (curve 3) QSC ($\dot{\varepsilon }$ = 10^−3^ s^−1^) and (curve 4) high-strain-rate SHPB ($\dot{\varepsilon }$ = 1550 s^−1^) tests.

The X-ray diffraction patterns of TiC MMCs in the initial state, after the QSC and the high-strain-rate SHPB tests, are compared with the base Ti64BEPM material in Figure 7.

The as-sintered Ti64BEPM material is predominantly characterized by the presence of α-phase, and a very small amount of β-Ti (less than 5% by vol.), which is difficult to see and can be traced by the presence of {002}Tiβ peak (Figure 7, curve 1). The addition of TiC particles causes the appearance of the TiC phase diffraction, and a significant increase in peaks intensity of the α-Ti phase (Figure 7, curve 2). There is a slight shift in the position of the α-Ti peaks compared to the peaks of the base material without TiC, which is likely associated with some changes in the chemical composition of the titanium-based matrix. (As will be shown below (see Section 4.2, Figure 21, and Table 4), an interaction of titanium with TiC particles took place that leads to some depletion of the matrix with titanium.) The relatively weak peak {002} of β-phase becomes distinguishable. After both types of compression tests, the magnitude of all peaks decreased to approximately the level that existed for the initial sintered state (curves 3 and 4). There was no broadening of the peaks observed after both, the quasi-static and dynamic compression tests. Such observation suggests that [39] there has been no accumulation of residual elastic stresses in either the α-phase or the TiC particles. The whole deformation energy dissipates probably by crushing the hard particles and plastic flow of matrix phases and formation of a dislocation substructure in therein. There are no significant changes in the crystallographic texture of the samples after both types of compression tests used, as shown by the (102) pole figures of α-Ti (compare Figure 4d,f with Figure 4c).

**Figure 8:**
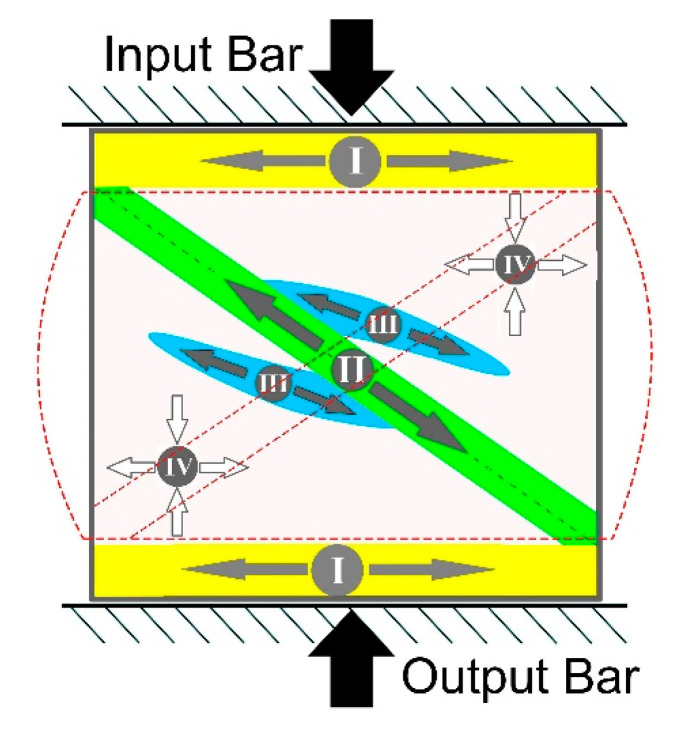
Schematic map of the location of the different zones in the longitudinal section of the test cylindrical samples. Roman numbers denote a certain zone. Black arrows indicate the direction of compression force.

Certain zones outstanding in the direction of compressive force in the longitudinal section of the tested cylindrical samples during the SHPB and the QSC compression tests were determined in a previous study performed on an alloy Ti64 with different microstructures [22]. The resulting microstructures were quite explicit for the identified zones where the stresses act differently on parts of tested sample (Figure 8). It has been shown that the deformed structure results from the action of the high-strain rate compression depending on the type and dispersion of the original microstructure [22].

**Figure 9:**
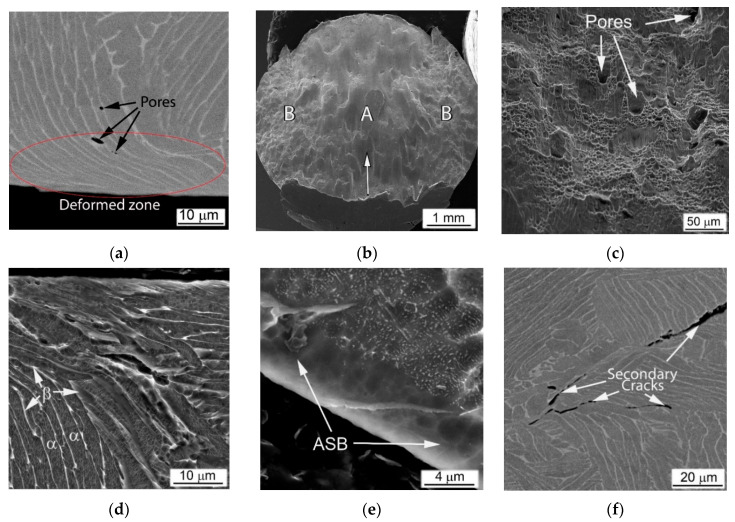
Microstructure (**a**,**d**–**f**), and fracture surface (**b**,**c**) of Ti64BEPM specimens tested with the strain rates: (**a**) 2220 s^−1^ (not broken), and (**b**–**e**) 2390 s^−1^ (broken). (**a**,**d**) Zone I, (**e**) Zone II, (**f**) Zone III. SEM, (**a**,**f**) SE, (**b**–**e**) BSE. The specimens were compressed along the vertical axis as shown in Figure 8, and images in (**a**,**d**–**f**) are aligned with their vertical axis along with the acting external load like in Figure 8.

Suggested implication is entirely true for the present evaluation of the metal-matrix composites obtained using BEPM. The Ti64BEPM alloy has been studied in details in this earlier work and it would be useful to assess the structures obtained in present study similarly, to specify the following important arguments. The samples Ti64BEPM showed signs of plastic deformation mainly in Zone I, i.e., in the areas of contact with the surfaces of input and output bars (Figure 1). This deformation zone was most evident on non-fractured samples and was obviously near the specimen contact surface with the front face of the input bar (Figure 9a). Collapsed residual pores were also observed at various locations across the sample (Figure 9a).

The compression applied at higher strain rate (with 2390 s^−1^) causes samples to fracture. The fracture surface is characterized by a typical rectilinear zone, which indicates the main crack propagation (A arrowed in Figure 9b) and adjacent to it zones of the secondary cracks spread (B, ibid). A higher magnification images reveal additional features, namely, residual pores (Figure 9c), generally absent in the cast and wrought alloy [22,39,40]. The microstructure of such Ti64BEPM specimens was quite similar to the alloy Ti64 with a lamellar structure, in which a plastically deformed Zone I has approximately the same depth close to 20–30 μm (Figure 9d). The main crack initiates and propagates within the Zone II running through the entire cylinder at an angle of about 45° to its vertical axis. This zone is a very narrow layer of adiabatic shear band (ASB), which has a thickness of about 5–6 μm near the edge of the cylinder (Figure 9e). The smaller secondary cracks associated with β-grain and α-colony boundaries are observed in the Zone III (Figure 9f). Specific evidence of plastic deformation was not found within Zone IV, while fine α-needles were observed inside individual α-lamellae and identified as α’-martensite (details are discussed elsewhere [22]).

**Figure 10:**
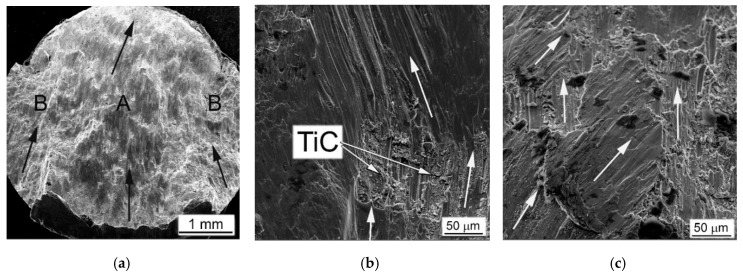

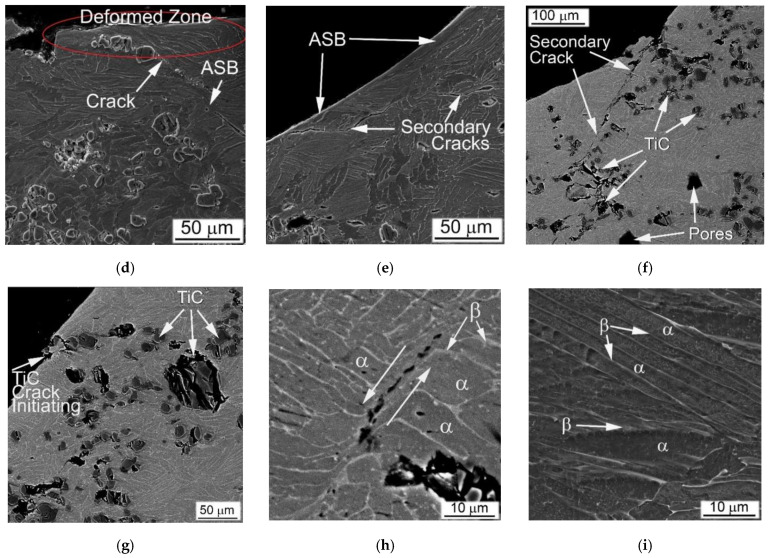
Typical fracture surface (**a**–**c**) and microstructure (**d**–**i**) of Ti64BEPM + 5TiC MMC SHPB tested at 2040 s^−1^ strain rate. Arrows in (**a**,**c**) indicates directions of cracks growth. (**d**) Zone I, (**e**–**g**) Zone II; (**h**) Zone III (**i**) Zone IV. SEM, (**a**–**d**,**g**,**i**) SE, (**e**,**f**,**h**) BSE. (**f**,**g**,**h**) polished; (**d**,**e**,**i**) polished and etched. Images (**d**–**i**) are aligned with their vertical axis along with the acting external load like in Figure 8.

The MMCs containing TiC particles were fractured at relatively lower strain rates, and their fracture morphology and microstructure differed significantly compared to the base Ti64BEPM material. The representative microstructures of Ti64BEPM + 5TiC are shown in Figure 10. During the fracture, the growth direction of the main (A in Figure 10a) and peripheral (B, ibid.) cracks differs in this composite from the base BEPM alloy (without TiC). As can be seen the main crack can actually change the direction of its further propagation when meets the clusters of particles TiC (Figure 10b). Similarly, the change in the initial direction of the crack growth is observed for smaller secondary cracks (Figure 10c).

The deformed Zone I was less pronounced in MMC than the Ti64BEPM TiC-free (Figure 10d vs. Figure 9a). Rather thin, not more than 5–7 μm, ASB was observed in Zone II in some places free of TiC particles close to the main crack (Figure 10e). In most places where TiC particles lie on the fracture surface, the ASBs were not observed. Moreover, these particles not only changed the direction of the crack propagation, but also served as initiators of secondary cracks (Figure 10e,f). The majority of the residual pores appear unchanged (Figure 10f), but particles TiC, especially larger particles or aggregates of smaller ones, break down (Figure 10g). The microstructure of the matrix alloy in the Zone III shows signs of quite strong plastic deformation, including shear displacement inside the α + β packets within some grains, probably with some involvement of residual pores in this process (Figure 10h). Finally, no evidence of deformation was found in Zone IV (Figure 10i).

**Figure 11:**
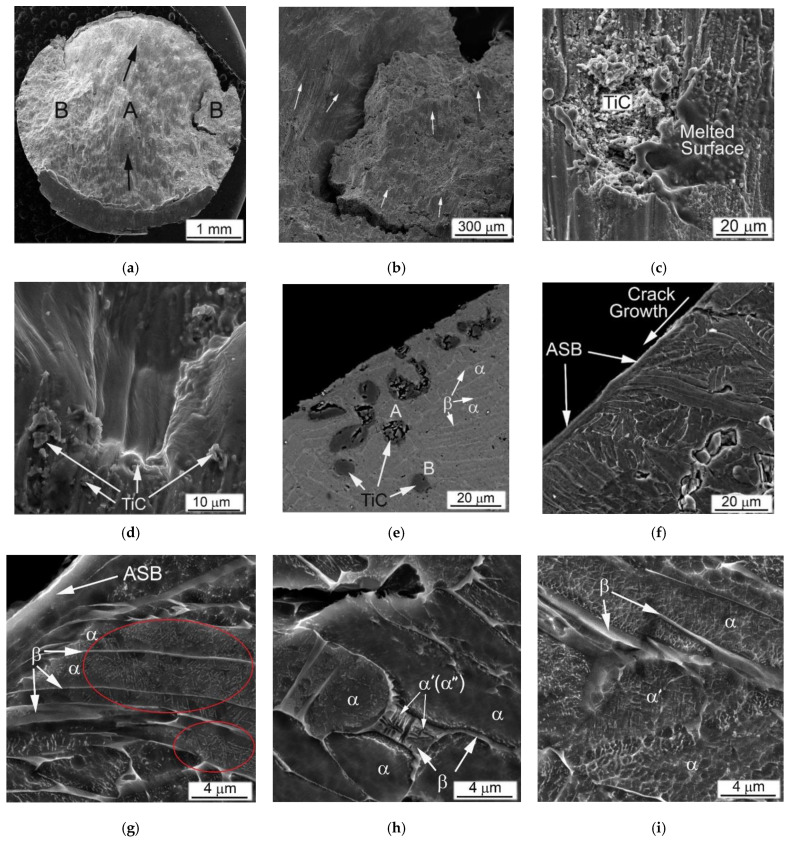
Fracture surface (**a**–**d**) and microstructure (**e**–**i**) of Ti64BEPM + 10TiC, SHPB tested with rate 1640 s^−1^. Arrows in (**a**,**d**) indicate the direction of cracks growth. (**e**–**g**) Zone II; (**h**,**i**) Zone IV. Ovals in (**g**) indicate the areas within α- lamellas containing martensitic needles. The broken TiC particles near the main crack are A labeled in (**e**), while not broken particles a bit away from the main crack are B labeled. SEM, (**a**–**d**) (**f**–**i**) SE, ((**e**), polished) BSE; (**f**–**i**) polished and etched. Images (**e**–**i**) are aligned with their vertical axis along with the acting external load like in Figure 8.

The MMC with a higher content of TiC particles after the high-strain-rate test demonstrates a similar fracture surface (Figure 11a–d) and microstructure (Figure 11e–i), as discussed above. As in the previous case, the propagation directions of the main and peripheral cracks passing through the coarse TiC particles and their agglomerates have changed (Figure 11a), but a more distinct secondary cracking has occurred (Figure 11b). Several cases of melting of small areas on the fracture surface were found (Figure 11c). Their presence obviously implies significant local heating, which can be observed even for specimens deformed at this relatively slow strain rate (1640 s^−1^). In Ti64BEPM + 10TiC, similar to 5% composite, carbides strongly affect the propagation of cracks, although the fracture character within the matrix alloy stays ductile (Figure 11d). Alike the former case, carbide particles, especially larger ones, which are located near the main crack, were broken up (Figure 11e), and thin ASB was observed near the crack surface free of TiC particles (Figure 11f).

Remarkable structural features have been found in this sample at various locations within Zones II, III, and IV shown in Figure 11g–i, respectively. They resemble a highly dispersed needles, mutually oriented to each other at an angle of about 60°. Previously, such structures were reported for Ti64BEPM, as well as for the cast and wrought Ti64GL after SHPB tested at much higher strain rates, namely, 2390 s^−1^ and 3330 s^−1^, respectively [22]. The observed structures were explained as α’-martensite formation inside untransformed broad α-lamellae. The occurrence probability of such a transformation was explained due to the dynamic loading conditions, which cause local rapid heating of relatively small samples, and then their rapid cooling, resulting from heat dissipation into SHPB metal bars with relatively good thermal conductivity. In this case, two types of martensitic needles with slightly different dispersion have been found, i.e., more dispersed as shown in Figure 11g and somewhat coarser as can be seen in Figure 11h. The chemical microanalysis data presented in Table 3 show that the coarser martensitic needles had a higher content of the β- stabilizing elements of vanadium and iron, while, in contrast, α- stabilizing aluminum was present in a smaller amount (p. #2 vs. p#1 in Table 3). The observed increased content of β-stabilizers should lead to the formation of orthorhombic α’’- martensite rather than hexagonal α’- martensite [1,41,42,43,44]. Most likely, this occurs due to some cooling delay within the local micro-volumes, which leads to the redistribution of alloying elements between the β- and α-phases, enriching the last phase with vanadium. There is also the possibility of some micro-inhomogeneity of the matrix alloy, which was formed during the synthesis of MMC. The second explanation can also be supported by the earlier discovered ability of partial carbon diffusion from titanium carbide particles into the matrix alloy during sintering [32,35,36]. This mechanism undoubtedly leads to a certain diffusion redistribution of titanium and alloying elements between phase constituents. In a certain sense, it may seem surprising that this phenomenon (formation of martensite) has been observed in α-lamellae of Ti64 alloys free of hard particles at least twice the strain rates than in the present Ti64BEPM + 10TiC MMC. It is also obvious that the presence of solid particles TiC in the structure during intense plastic deformation can cause additional localization of the plastic flow even at lower rates, since these particles play the role of additional local stress concentrators, which in turn can cause more intense local heating.

**Figure 12:**
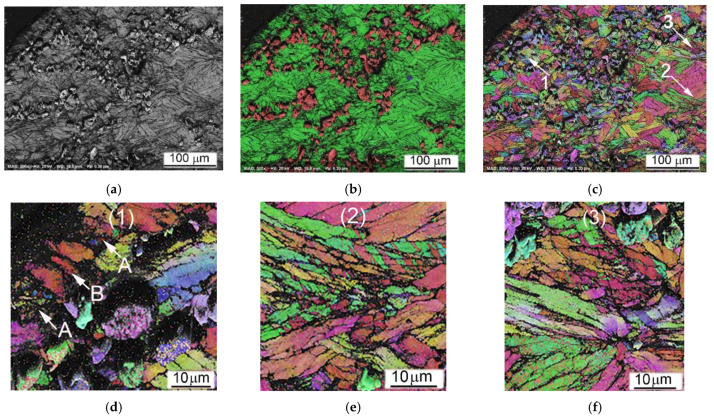
SEM EBSD images of the fractured composite Ti64BEPM + 10TiC after SHPB test with the rate 1640 s^−1^. Pattern Quality Map (**a**)**,** phase map (**b**), and orientation map (**c**–**f**) images. The α-phase is shown in (**b**) using green, β-phase blue, and TiC red colors. All images are taken in vicinity of the Zone II (Figure 8); the images (**a**–**c**) centers are within 200 μm away from the place of fracture (main crack) visible in the left top corner of the images (**a**–**c**). The numbered areas in (**c**) are shown at higher magnification images: (1) in (**d**), (2) in (**e**) and (3) in (**f**).

Important conclusions on the deformation mechanism can be drawn from EBSD results revealing the local crystal structure and some of its defects. The images presented in Figure 12 show the local microstructure of the cylindrical sample Ti64BEPM+10TiC fractured along Zone II (Figure 8) during the SHPB test with the strain rate 1640 s^−1^. The fractured surface (cross-section) can be seen in the upper left corner of Figure 12a–c. In an earlier study of the SHPB tested Ti64 alloy, acceptable EBSD quality patterns/results were obtained for lamellar microstructure samples deformed only at relatively low strain rates [22]. The pattern quality map (a) shows regions of darker gray levels in the vicinity of the TiC particles indicating that these regions contain significantly larger dislocation densities than the rest of the sample covered in this EBSD map. The high dislocation density decreases the local crystallinity state, therefore these regions appear darker in the pattern quality map and non-indexed in the phase and orientation maps. These results are very consistent with the expected increased strain rate seen by the matrix in the vicinity of the TiC particles. The structure adjacent to the fractured surface depicted in Figure 12d shows that some of the α-phase lamellae become highly fragmented (A-labeled) with the submicron blocks’ size, while some other lamellae (B-labeled) just next to them practically remain unchanged. The structure at a distance within of several hundred microns from Zone II is also very heterogeneous, especially near particles TiC or the α-lamellae colonies boundaries or β-grains boundaries. For example, lamellae are finer and more deformed close to particles TiC, multiple twinning in the α-lamellae can be seen close to TiC particles and boundaries of β-grains as shown in Figure 12e. Areas of larger plastic deformation in some lamellae can be seen away from Zone II (about 300 μm), as shown by a large green lamella at the center of Figure 12f. The change of orientation of this lamella is about 20 deg. within 10 μm space and its tip close to particle TiC is highly deformed, whereas the rest of lamella is deformed only slightly. The reasons of observed high heterogeneity of plastic deformation are obviously related to the existing structural defects, such as: particles TiC, β-grains boundaries, and α-lamellae colonies boundaries. However, high deformation heterogeneity can be also resulted from effect of orientation of lamellae toward the acting load, favoring dislocation motion mechanism, subblocks building, twinning, etc.

**Figure 13:**
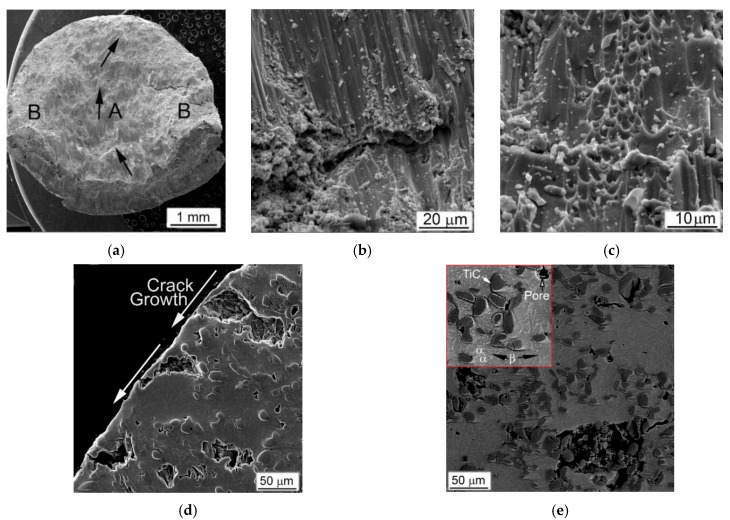
Fracture surface (**a**–**c**) and microstructure (**d**,**e**) of Ti64BEPM + 20TiC sample, SHPB tested with rate 1470 s^−1^. Arrows in (**a**,**d**) indicates directions of cracks growth. (**d**) Zone II; (**e**) Zone IV. SEM, (**a**–**c**) SE, (**d**,**e**) BSE images.

In general, the fracture surface and internal microstructure of the SHPB tested Ti64BEPM + 20TiC samples (Figure 13) were very similar to above discussed results for the composites containing smaller amounts of titanium carbide particles. The growth directions of the main and peripheral cracks changed during the high strain-rate compression (Figure 13a). Carbide particles affect the direction of cracks propagation not only in the plane propagation of the main crack, but also at an angle to it (Figure 13b). A plurality of dimples can be seen inside the titanium-based matrix, which are typical of the ductile fracture, but a large number of carbide particles splinters are also observed on the fracture surface, which are obviously result of the crack spread (Figure 13c). The internal microstructure of the sample had quite distinctive features. In the immediate vicinity of the surface of the main crack, as well as away from it, only large TiC agglomerates were destroyed (some of them felt out of during samples preparation because of relatively poor bond with matrix), while several smaller individual particles had some cracks (Figure 13d,e).

The above results suggest that the presence of hardening particles TiC, even in such small amounts as 5%, plays a critical role in the deformation and fracture of the MMC. The hard (but brittle) particles are broken under the applied impact load and predetermine the deformation and damage of the Ti-based alloy matrix. From a comparison of the results obtained for the Ti64BEPM alloy and MMCs containing 5% and 10% TiC, it can be concluded that the presence of the particles and an increase in their content leads to the switching of the “weakest” microstructural element responsible for the bulk of the *SE* dissipation: from purely plastic flow (deformation) of the matrix to brittle fracture of the TiC particles that initiate the onset of cracks and promote their propagation. In the Ti64BEPM alloy, in the absence of TiC, the role of the “weakest link” is played by the boundaries of β- grains covered by coarse α-lamellae and the boundaries of α + β colonies (Figure 6f) already discussed in more details elsewhere [22]. The introduction to Ti64BEPM alloy of particles of TiC can change the nature of the mechanism of deformation and fracture. At 5% of the carbide particles TiC, which mainly caused an increase in the strength of the material (compare Figure 5a with Figure 5b, and Curves ## 1 and 2 in Figure 6a), and only when the strain rate reached $\dot{\varepsilon }$_cr_ value, most of the particles begin to break, thereby causing the formation of cracks fracturing the specimen. The structure embrittlement effect grows with further increase of TiC portion within the MMC resulting in decrease in V_cr_ at which material cracks. For example, at 20%TiC the reserve of plasticity of Ti64 matrix is insufficient for prevention of destruction of material even at quite low strain rate such as 1470 s^−1^, not talking about higher rates (Figure 5d, and curve #4 in Figure 6).

**Figure 14:**
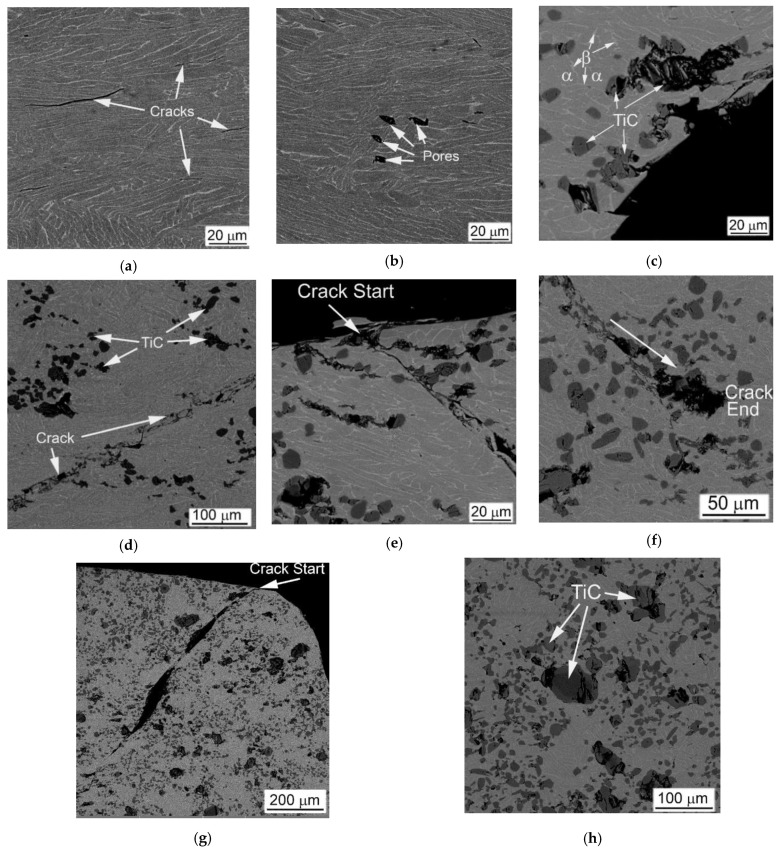
SEM images of samples after QSC tests with rate 10^−3^ s^−1^: (**a**,**b**) Ti64BEPM, (**c**,**d**) Ti64BEPM + 5TiC, (**e**,**f**) Ti64BEPM + 10TiC, and (**g**,**h**) Ti64BEPM + 20TiC. (**a**,**b**,**d**,**h**) Zone IV, (**c**) Zone II, (**e**,**g**) Zone I, and (**f**) Zone III. BSE.

The particles of TiC have a very similar effect on the mechanical behavior in QSC condition. The introduction of 5%TiC in Ti64BEPM resulted in a slight increase in strength as well as a slight decrease in plasticity, as seen from comparison of curves #4 in Figure 5a,b. The strength level remains rather the same at 10% and 20% of TiC, while the plasticity reduction becomes more evident (Figure 5c,d). Such differences in mechanical behavior are directly related to the specifics of the deformation mechanism, which is affected by the microstructure of the specimens (Figure 14). The Ti64BEPM structure after QSC tests was characterized by clear signs of uniform plastic deformation throughout the entire sample volume (Figure 14a). At the same time, small cracks were also observed at various places of interfacial α/β- boundaries, or at the boundaries of α-Ti colonies. In turn, the residual pores, they mainly collapsed (at least partially) without cracks initiation (Figure 14b).

The situation changes drastically in the presence of TiC particles. First of all, all samples, as a rule, are destroyed by the main crack propagating at an angle of about 45° to the applied load (Figure 14c). There are numerous secondary cracks appears in addition to the main crack. (Figure 14d–g). The carbide particles located on the surface of the specimens can initiate these cracks (Figure 14e,g), while the particles inside the specimens can stops the cracks propagation (Figure 14f). At the same time, predominantly large TiC particles and their conglomerates breaks (Figure 14c,f,h), while the small particles remain almost unchanged. The signs of plastic flow are observed in the matrix of composite only at 5% and 10% of TiC particles (Figure 14d,e).

#### 4.2.2. MMC Reinforced with TiB

**Figure 15:**
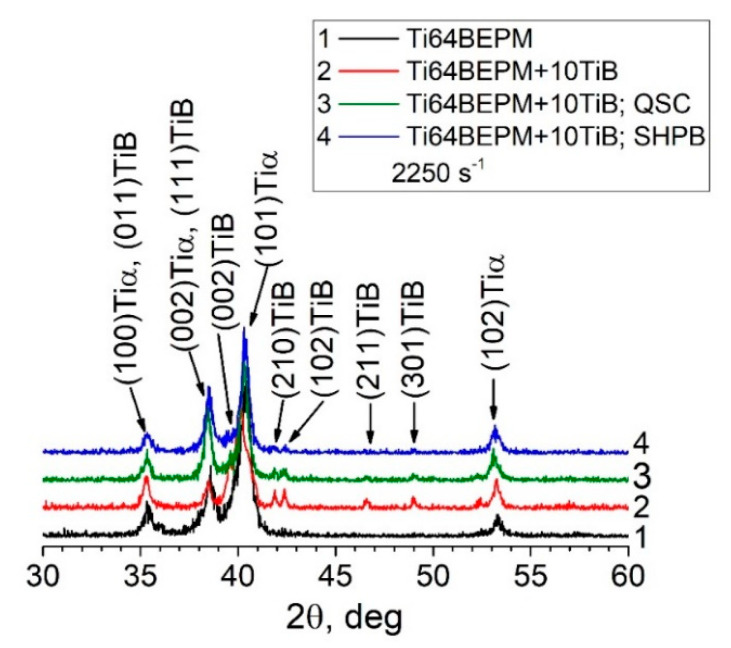
The X-ray diffraction patterns of (curve 1) Ti64BEPM, and Ti64BEPM + 10TiB in (curve 2) as-sintered state, and after QSC ($\dot{\varepsilon }$ = 10^−3^ s^−1^) (curves 3 and 4), and SHPB ($\dot{\varepsilon }$ = 2250 s^−1^) tests.

A comparison of the X-ray diffraction patterns of the base Ti64BEPM alloy and its composite reinforced with 10% particles of TiB in the sintered state, as well as after deformation at different strain rates is shown in Figure 15. The formation of TiB phase during sintering is reflected by the presence of corresponding X-ray peaks in the diffraction pattern, which are particularly well resolved on untested specimens (compare curves 1 and 2 in Figure 15). These peaks become difficult to distinguish for specimens deformed under quasi-static and high strain-rate compression (curves 3 and 4, ibid.).

The textures of the α-phase crystallites of these composites after both types of compression are more distinct than the initial states after sintering (including Ti64BEPM + XTiC), showing a relatively higher (102)α pole density at the center of the pole figures (Figure 4e,g vs. Figure 4c).

**Figure 16:**
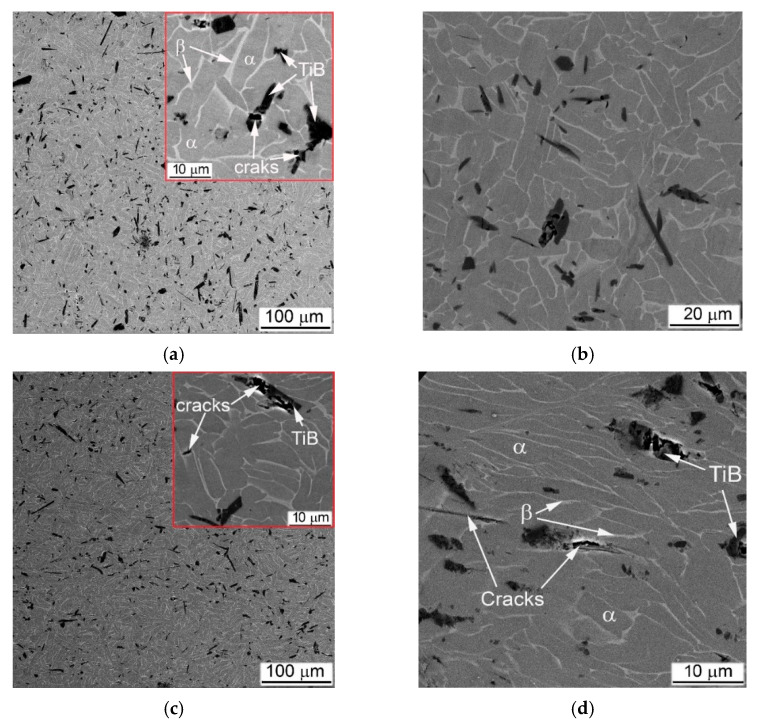
SEM BSE images of Ti64 + 5TiB after the high-strain-rate SHPB test with rates (**a**,**b**)—1430 s^−1^, (**c**,**d**)—2930 s^−1^. (**a**,**c**)—Zone II; (**b**,**d**)—Zone I.

As noted earlier, MMC strengthened by TiB particles did not break down during high strain-rates compression tests. This behavior was adequately reflected in the assessment of microstructure after compression tests. There was no evidence of significant plastic deformation within α + β- matrix found by SEM for 5% of TiB composites, even in areas within cylinder samples where deformations were expected to be greatest, such as Zones I and II; as was no observed either ASB or intensive cracking (Figure 16). The only signs of deformation were cracks in TiB particles (Figure 16a,c), which at higher strain rates also spread to the α + β- matrix (Figure 16d). In fairness, some elongation of the α + β packets as a result of the plastic flow can be noted at higher strain rates, as shown in Figure 16d.

**Figure 17:**
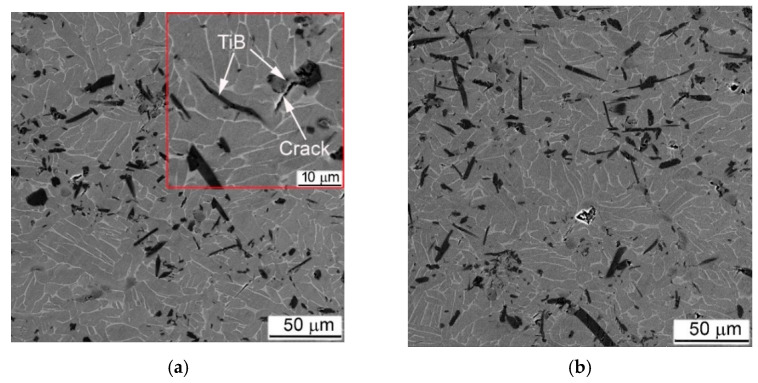

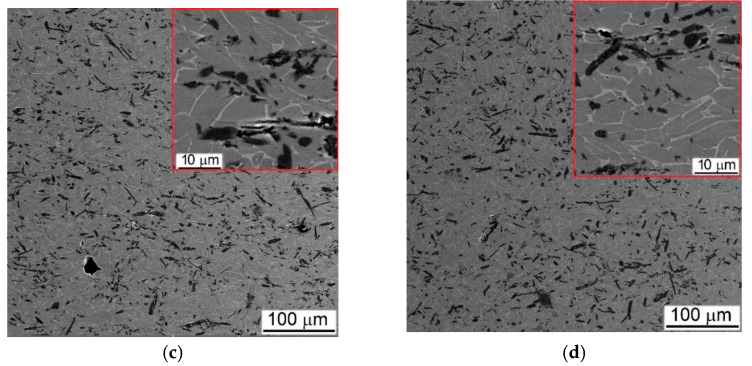
SEM BSE images of Ti64 + 10TiB after the SHPB tests with strain rates (**a**,**b**)—1280 s^−1^, (**c**,**d**)—2510 s^−1^. (**a**,**c**)—Zone II; (**b**,**d**)—Zone I.

The structure of 10% of TiB MMC after the high strain-rates test also did not show noticeable signs of plastic deformation in α + β-matrix (Figure 17). As for Ti64BEPM + 10TiB, SEM finds mainly cracking of TiB lamellae (Figure 17a,c). There was also no principal difference observed between the structures of Zones I and II, and no differences after a twofold strain rate increase as shown in Figure 17a,b vs. Figure 17c,d. Such “microstructural resistance” of MMCs reinforced by particles of TiB to high strain-rate compression—in contrast to TiC MMCs—explains the stability of the strain energy characteristics with increasing the strain rate (curves 5 and 6 vs. curves 2 and 3 in Figure 6a).

**Figure 18:**
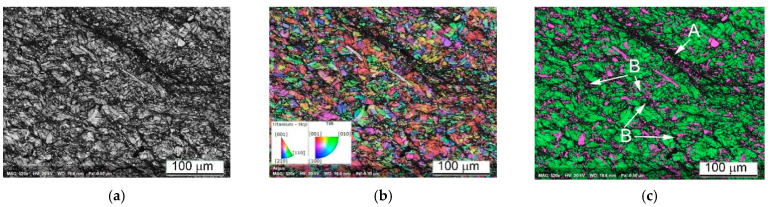

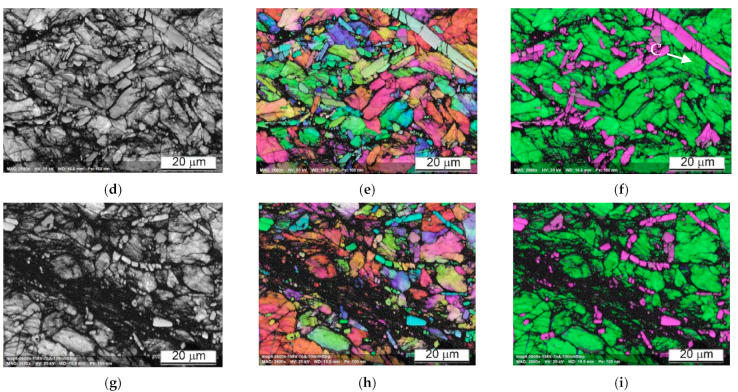
SEM EBSD images of Ti64BEPM + 10TiB sample after SHPB test with rate 2510 s^−1^. The sample stays unbroken after the test, but forms the ASB area in the Zone II (Figure 8). Pattern Quality Maps (**a**,**d**,**g**); orientation maps (**b**,**e**,**h**) phase maps (**c**,**f**,**i**) images. The green color in phase map images represents α-phase, blue β-phase, and magenta TiB particles.

The EBSD data of Ti64BEPM + 10TiB sample after SHPB test are shown in Figure 18. While this sample maintained macroscopic integrity after the test, the EBSD results indicate that the sample experience localized shear deformation at the microscopic level. The dark band labeled A in Figure 18c shows a major shear band where a much higher dislocation density has lowered the local crystallinity of the matrix resulting in a high ratio of non-indexed points. Most of the few points indexed inside this shear band are from the TiB particles which were less affected by the severe plastic deformation due to their much higher hardness. Additional, minor shear bands, labeled B in Figure 18c, occur similarly in the vicinity of particles TiB. EBSD observation also reveals the fragmentation of the large TiB particles. However, conventional EBSD technique has difficulty to fully resolve the β-Ti phase expected in this alloy and that is obviously due to the sub-micron dimension of this phase regions. Nevertheless, some larger β-Ti lamellae can be seen, for instance, the one labelled C in Figure 18f. Resolving the small or highly deformed crystals was even harder after the SHPB test when the structure refined to domains smaller than the spatial resolution of conventional EBSD technique. This was one of the reasons to use the Transmission Kikuchi Diffraction (TKD) technique to investigate these microstructures at the nanometer scale to confirm the crystallinity of shear bands and very fine α- and β-Ti grains that have been modified beyond the resolution reach of conventional EBSD.

As can be seen from Figure 18, despite the absence of the cracks, it was possible to identify a band of severe plastic deformation, A and B labeled in Figure 18a–c. It can be noted that all these bands of localized plastic deformation seem to evade microvolumes with large particles of TiB. At higher magnification, it becomes clear that the TiB particles have no internal defects. The largest particles break up into individual fragments, while the many smaller ones seem to retain their original shape and size (Figure 18d–f). Dislocations density (affecting pattern quality in EBSD and TKD maps) inside the α-phase lamellae seems increased (Figure 18g). Intense plastic deformation is localized in microvolumes surrounding TiB particles, and these areas look like dark regions with poorly resolved Kikuchi patterns obscuring a clear orientation map (Figure 18g–i).

**Figure 19:**
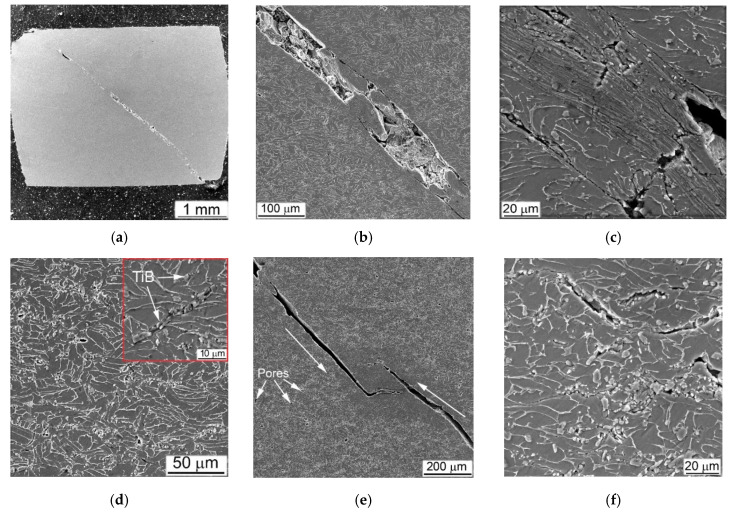
SEM images of (**a**–**d**) Ti64BEPM + 5TiB, and (**e**,**f**) Ti64BEPM + 10TiB after QSC tested with the rate 10^−3^ s^−1^. (**a**) General view of the specimen, (**b**,**c**,**e**) Zone II, (**d**,**f**) Zone III. SEM, (**a**) SE, (**b**–**f**) BSE. Large arrows in (**e**) indicate the shift direction of the sample’s halves.

TiB MMC specimens deformed with a quasi-static strain rate show a macroscopic cracking, in contrast to dynamically loaded samples (Figure 19). That was the obvious result of essentially higher general strain achieved (curves 4 vs. curves 1–3 in Figure 5e,f). After quasi-static loading, both composites strengthened with TiB formed a main crack crossing samples at an angle 45° to the main axis of the cylinder (Figure 19a). In this case, the main plastic deformation was located exactly near the main crack (Figure 19b,c). All other regions remote from the crack were characterized with practically uniformly deformed structure. The specimen deformation was mainly caused by crushing the TiB particles (Figure 19d). Comparing the samples with 5% and 10% of TiB (Figure 19a,d), it can be concluded that such a wide (up to 100 μm) band of cracks crossing the samples was formed by merging two separate cracks growing from opposite ends of the cylindrical samples. The QSC test showed more intense cracking of the TiB particles at a higher their content, which in turn caused more intense secondary cracking of the matrix phase (Figure 19f). Comparison of the matrix structure of the alloy Ti64BEPM + TiB in the initial state (Figure 2e,f), with its structure after the QSC test (Figure 19d,f) reveals noticeable differences resulting from plastic deformation, while the similar comparison of the structures after SHPB tests (Figure 17b) does not show significant changes in most zones.

**Figure 20:**
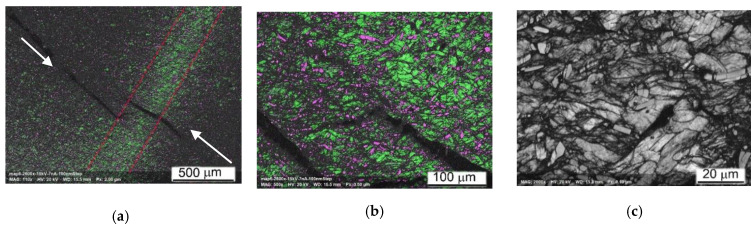

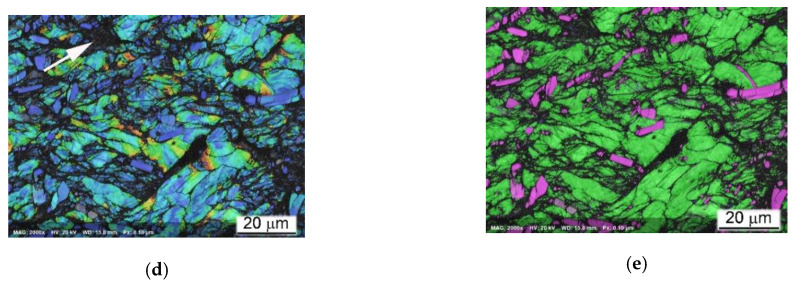
SEM EBSD results of Ti64BEPM + 10TiB sample after QSC tested with rate 10^−3^ s^−1^. The shown area is the same as in Figure 19e. Phase map images (**a**,**b**,**e**), where α-phase is shown in green, β-phase in blue and TiB in magenta. (**c**)—Pattern Quality Map. (**d**)—Grain Misorientation Map.

EBSD results shown in Figure 20a,b indicates the presence of two large cracks (arrowed in Figure 20a) surrounded by regions containing very fine grains and a high fraction of non-indexed points which are the key indicators of severe plastic deformation associated with localized shear. Furthermore, the two cracks seem to have been in the process of merging when the quasi-static deformation process stopped. It is likely that the strain rate experienced by this sample was just below the threshold value that would have resulted in a catastrophic break-up. As in the case of a high strain-rate deformation, the TiB particles loaded quasi-statically were also fragmented. The TiB fragments were additionally bent by the plastically deformed α-crystals with well-defined orientation, and showing developed dislocation substructure (Figure 20d,e).

Figure 20 demonstrates very good correlation between the predicted deformation pattern and that experimentally observed. It shows the crack formation exactly along Zone II (Figure 8), of predicted maximum stress zone. It also can be seen that the experimentally observed load distribution is slightly more complicated than predicted, and EBSD results prove this. We see an area, a green stripe in (highlighted by red dashed lines in Figure 20a), where the plastic deformation was lowest. This stripe region is denoted by a much higher indexing rate than the rest of the map, the grey regions being not indexed due to a weak diffraction signal or too fine structure. It propagates in a direction close to the orthogonal direction to Zone II; however, for confirming this statement, a further sectioning of the sample should be also considered. Nevertheless, it is most likely that the entire strain localization feature runs diagonally across the sample, and it is probably more correctly show its propagation in the sample from corner to corner, as it is sketched with red dashed lines in Figure 8; Figure 20a. It becomes evident that the main crack is the result of two cracks formed simultaneously: one is at the contact with the Input Bar of the sample top and the second is at the Output Bar contact in the lower part of the cylinder sample. Since two cracks meet right in the middle of the cylinder, it suggests that they form approximately simultaneously. It also suggests that Zone II should have been formed at a very specific angle. This angle corresponds to the typical 45-degree deformation localization in compression test, which is valid to most materials, such as metals, plastics, etc. toward the main axis of the cylinder. Thus, we can only expect to see one crack if a certain ratio between the height and diameter of the cylinder is held. This sample was a successful combination of test parameters as it shows the onset of cracking at both ends and their encounter in the center. If the strain rate were slightly higher, the sample would break in two, and it would be less convincing or easy to reach this conclusion. The fact that the two cracks are misaligned by ~100 μm may be due to the shape ratio of the initial undeformed sample (not having an ideal square cross section) or to the presence of TiB particles which may divert the crack propagation slightly from the ideal 45 degrees tilt from the cylinder axis. The higher magnification orientation maps explain what the darker regions in (a) and (b) represent. There are areas of highly fragmented and plastically deformed matrix alloy structure. One of such area is marked by an arrow in (d). Crystals are fragmented into sub-100 nm domains and are very difficult to resolve by conventional EBSD. These questions can be answered by orientation mapping in transmission mode (TKD in SEM). The image (d) also shows that some grains have experienced a relatively high deformation. Despite the grains’ size seeming to not have changed significantly from their initial state, the EBSD results reveal lattice rotations of 15–20 degrees within a few micrometers across the same grain, which in the context of QSC process, can be considered as relatively high values. The grains of the Ti64 matrix alloy demonstrate a developed dislocation system. The smaller particles of TiB were unaffected by the deformation process (two magenta needles in the center of (e)). The TiB particles ~5 μm and larger become fragmented with no signs of plastic deformation. It clearly defines two different ways of dissipating the applied load energy: plastic deformation inside the ductile matrix and structure fragmentation inside the brittle reinforcement particles.

**Figure 21:**
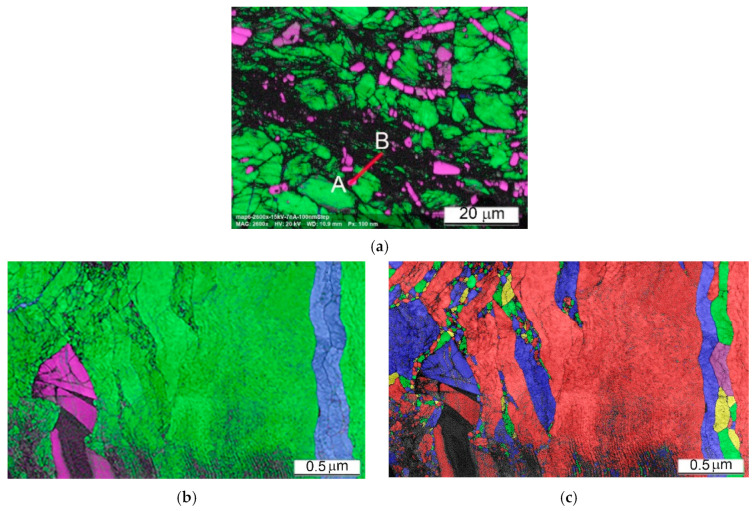

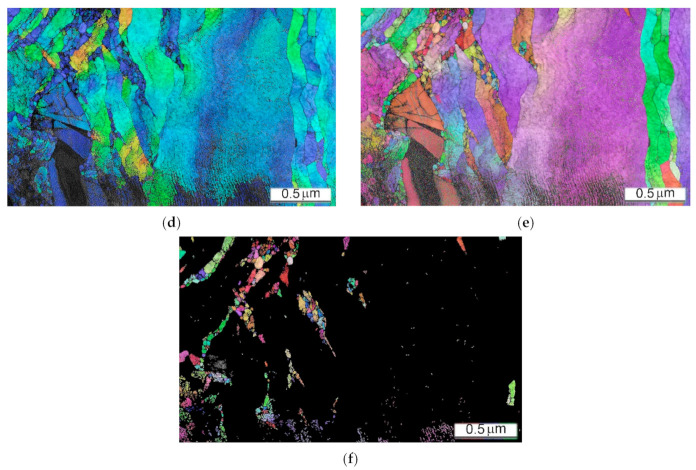
TKD results of the heavily deformed region (ASB area) on the sample Ti64BEPM + 10TiB after SHPB test with the strain rate 2510 s^−1^. EBSD (conventional) phase map (**a**) showing the area from where the lift-off sample (AB section) for TKD was taken; within α-phase is shown in green, β-phase in blue and TiB is in magenta colors. The rest of the images (**b**–**f**) show TKD results: phase map (**b**), similarly color coded as in (**a**); grains map, in random color (**c**); GAM map (**d**); IPFz (**e**); IPFz map showing the grains <100 nm (**f**).

The lift-off sample for TKD was cut with focused ion beam system from the ASB region of the Ti64BEPM + 10TiB sample after the SHPB test with the strain rate 2510 s^−1^ as shown in Figure 21a with a red AB section. The region of interest was first identified using the conventional EBSD as shown in Figure 21a, allowing the detection of physically distinct structures within the ASB. The section had a width of about 10 μm and a depth of about 5 μm and from point A to point B it covered four distinct structures:

(1) TiB particle (magenta);
(2) Relatively preserved, but most likely plastically deformed α-grains (green);
(3) ASB I is a region that is not resolved by conventional EBSD due to extremely high fragmentation of the initial α-grains and/or high dislocation density reducing pattern quality, as well as a region of possible recrystallization;
(4) ASB II is a region of a less deformed initial α-grains structure or possibly a region with a relatively larger recrystallized α-grains.

It is important to note that based on the resolution limit of the field-emission gun SEM-EBSD, the dimension of the crystallites not resolved within the ASB I area is 20–30 nm and smaller [45]. For the same reason, most β phase regions are not resolved by conventional EBSD, except for very few larger β-lamellae. In contrast, the β-phase lamellae are well resolved in the TKD images performed with 3 nm steps and shown in Figure 21b. Orientation maps shows highly heterogeneous plastic deformation in the ASB region. The α-grains experienced the highest deformation level in the vicinity of the TiB particles.

The Grain Average Misorientation (GAM) map shows misorientation of some grains around the TiB particle up to 20+ degrees in the 0.5 μm scale range (color changes from blue to orange), while at a distance of few microns from the particle the internal grains’ misorientation is usually twice as small (color changes from blue to green). Most importantly, recrystallization also begins in this area, due to extreme condition of the plastic deformation. In highly deformed sites surrounding the TiB particle, very small (nm) grains of α-phase are formed. Interestingly, the β-phase are also recrystallized. A small β-region on the upper left of the image (Figure 21b) clearly shows recrystallized β-grains. Very large recrystallized grains are not observed; therefore, it can be argued that a very early stage of recrystallization was found here. It can be noted that TKD results clearly show the different deformation mechanism of the structures involved. Brittle particles of TiB dissipate impact energy through fragmentation without signs of their plastic deformation, while crystallites of α and β-phase are plastically deformed.

### 4.3. The Effect of Type of Reinforcing Particles and Their Volume Fraction on Deformation Mechanism and Deformation Energy

**Figure 22:**
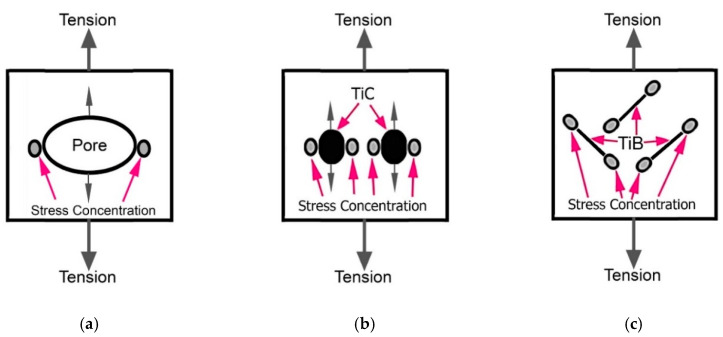

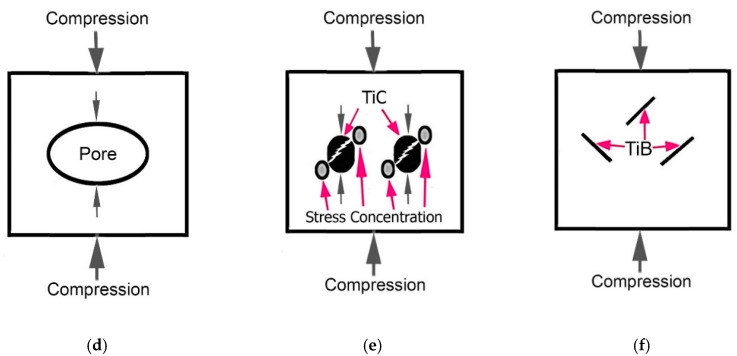
Schematic illustration of the stress concentration mechanisms involving pores (**a**,**d**), carbides (**b**,**e**), and borides (**c**,**f**) under tensile (**a**–**c**) and compression (**d**–**f**) test.

As shown above, the presences of the hard particles and pores has a significant effect on the deformation processes and mechanical properties of MMCs. It should also be emphasized that the presence of these structural elements has very different effect under tension test compared to compression (Figure 22). Both structural elements play the role of stress concentrators, primarily causing a drop in tensile ductility, which has been discussed previously [10,25,26,46]. This effect explains the decrease in the observed plasticity characteristics as shown in Table 2 and can be schematically illustrated as shown in Figure 22a,b. In case of the simultaneous presence of pores and particles TiC within the MMCs, the decrease in plasticity is more pronounced, due to their cumulative effect, resulting in fracture that obviously taking place in the region of elastic deformation rather far from reaching the values of Yield Stress (compare p. 1 with pp. 2–4 in Table 2). At the same time, these two structural elements have different effects of the stress concentration under compression. Residual pores collapse in the same way as in foam metals but with less pronounced effects on properties due to their relatively small fraction. While carbide particles break down, they initiate stress concentration at locations where cracks form in the metal matrix (compare Figure 22b,e). At the same time, TiB particles under tension cause a concentration of stress at the tips of their plates and/or needles, catastrophically embrittling MMCs (Figure 22c). However, with compression, such an effect is unlikely (Figure 22f). The suggested mechanisms allow to rationalize observed pronounced differences in mechanical properties: compare Figure 5b–d with Figure 5a, and p. 2–4 with p. 1 in Table 2.

The difference between the effect of TiC and TiB particles on the mechanical properties under tension and 3-point bending was discussed elsewhere [10,24]. Due to the needle or plate morphology TiB particles, it has been pointed out that, their sharp edges play more effective role as stress concentrators compared to the rounded edges of the TiC particles, especially when the plates or needles are specifically oriented relative to the applied external load, favoring an easy cracks nucleation. The presence of TiB particles has a less pronounced fracture embrittlement effect than TiC in the case of compression loading, especially at a high-rate strain. Obviously, this can be explained by the fact that titanium boride particles are formed in-situ by a chemical reaction forming particles of TiB with a shape far from equiaxial and a size varying over a wide range (Figure 3b). At the same time, the particles of TiC (Figure 3a) do not change their morphology and size within the matrix alloy during sintering or, as noted above, they tend to form conglomerates during mixing with the titanium hydride and the master alloy.

**Figure 23:**
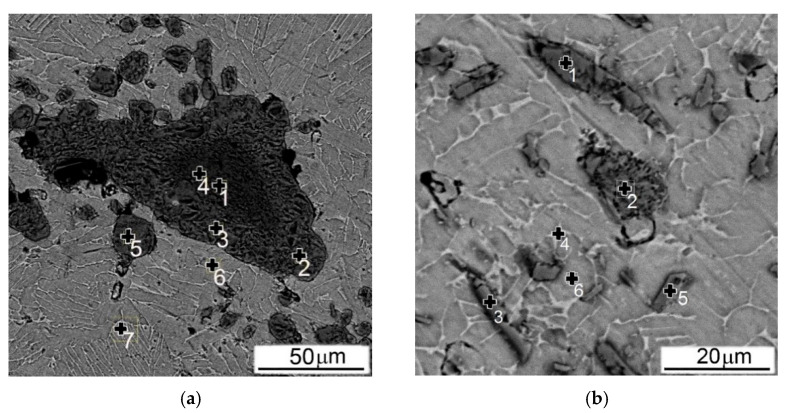
SEM images (BSE) of (**a**) Ti64BEPM + 10TiC and (**b**) Ti64BEPM + 10TiB with the marked locations where the EDS chemical composition point analysis was taken (see results in Table 4).

As a result, the particles size of TiB in both MMC varies in a rather narrow range: the thickness is 2–4 μm, and the length is 10–80 μm, while the particles size of TiC varies from several to hundreds of microns. In addition, the TiB particles are grown in-situ reacting with the surrounding Ti, and as a result have a stronger bond with the surrounding metal matrix. At the same time, globules of TiC partially joint into conglomerates, during mixing with metal components before pressing, and then during subsequent sintering, their interaction with the matrix metal is reduced only to a certain redistribution of carbon and titanium atoms between the matrix alloy and the particle without changing the shape and size of carbide inclusions [35,36]. Figure 23 shows examples of microstructures of MMCs with particles of TiC (Figure 23a) and TiB (Figure 23b) on which the chemical composition inside the particles and inside the matrix was measured. The results of these measurements are collected in Table 4.

As can be seen from the presented EDS data measured at the center of the coarse particle of TiC (or possibly the conglomerate of smaller ones at spots 1 and 4 in Figure 23a), the ratio between Ti and C is approximately 64/36 at. % (pp. 1 and 4 in Table 4), which does not correspond to the stoichiometric composition of titanium monocarbide. The peripheral portion of this particle is even more depleted with carbon (spots 2 and 3 in Figure 23a, and pp. 2 and 3 in Table 4). The smaller carbides have approximately the same carbon content as the periphery of the larger carbide: for example, spot 5 in Figure 23a and p. 5 in Table 4. As for the α + β matrix; its chemical composition is almost the same both in the areas adjacent to the carbides, and far from them (spots 6 and 7 in Figure 23a, and items 6 and 7 in Table 4, respectively). In addition, a very small amount of aluminum was found inside the carbide particles, while no vanadium was observed (items 1–5 in Table 4).

The chemical analysis carried out on particles of TiB within the composite showed that all particles of TiB, despite their size (spots 1–3 in Figure 23b), have a composition that does not correspond to the stoichiometric ratio and is rather enriched in titanium (pp. 8–10 in Table 4). It should be noted that unlike the above-mentioned case of titanium carbide, these borides have, in addition to a small amount of aluminum, also a minor vanadium presence. Another difference compared to MMC with 10% TiC is that in Ti64BEPM + 10TiB, small titanium-enriched particles can have relatively high aluminum content (spot 5 in Figure 23b and item 12 in Table 4). However, it is not high enough to claim the formation of titanium aluminide. It should be noted that α + β- matrix is boron free, supporting its good bonding with titanium boride (spot 6 in Figure 23b, and p. 13 in Table 4).

In other words, in the both cases of reinforcement with particles of carbides or borides, the particles are enriched with titanium, but depleted (relative to stoichiometric composition) with carbon and boron, respectively. At the same time, almost all boron goes to the formation of TiB plates or needles and is not found in the α + β matrix, while carbon quite actively diffuses into the titanium matrix.

The following viewpoints can be emphasized in the summary. Due to the processes involved in forming the structure of the composites during their sintering, the metal matrix composite strengthened with TiB particles is characterized by a relatively stronger bond between the metal matrix and the particles. In addition, TiB composites have relatively smaller particle sizes that do not exceed a few microns. Compared to the particles size in TiC MMC, when they sometimes reach several tens of micrometers, this is more than an order of magnitude greater than for borides. On a top of it, TiC particles tend to form relatively big aggregates of particles and form structural heterogeneity in the distribution of particles within the matrix. As a result, the Ti64BEPM + (X)TiB material is more resistant to high-strain-rate (dynamic) compression as opposed to TiC-containing analogs in a wide range of strain rates and is less prone to the formation of localized plastic deformation zones with the formation of adiabatic shear bands, followed by cracking. In a certain way, this is confirmed by data on sound frequency measurement and vibration damping (Table 2). According to these data, when the particles TiC are added into the Ti64BEPM matrix their fraction increase causes the Sound Frequency gradually increases, and the Damping increases with the particles TiC content raises up to 10%, and then falls (pp. 2–4 vs. p. 1, Table 2). If we compare MMCs containing TiB with those containing TiC, then for equal volume fractions of particles these characteristics in both cases will be higher for Ti64BEPM with TiB, namely: 13,624 Hz and 0.000284 versus 13,551 Hz and 0.000266 for 5% (p. 5 vs. p. 2, Table 2), and 14,219 Hz and 0.000322 versus 14,029 Hz and 0.000309 for 10% (p. 6 vs. p. 3, ibid); and, since the values of Young’s moduli remain almost equal for MMCs with an equal fraction of strengthening particles (compare pp. 2 and 3 with 5 and 6, respectively, Table 2), then according to [47,48], the above differences in response to the impulse excited vibrations will be more likely of all is due to the peculiarities of both the microstructure of the composites as whole, and the peculiarities of the interphase boundaries between the Ti64 matrix and solid particles inside it. The action of the latter interphase boundaries, which have a different nature than the α/β- boundaries present in the matrix, complements and enhances the action of the boundaries between the α- and β-phases, which play a decisive role in the formation of pores and cracks during deformation of the free of solid particles Ti64 alloy [22].

The above-mentioned features of MMCs hardened with different types of solid particles have a fundamentally different effect on the localization of deformation under high strain-rate loading. The TiB particles having much better bound to the matrix, and due to the significant difference with latter in elastic moduli, strength, and plasticity [10,49], as well as differences in their tribological properties [50], during deformation undergo intense friction with the flowing and enveloping the broken fragments of TiB particles matrix [23]. This friction causes generation of additional intense heating at the TiB/α + β interfaces. Moreover, an interface between dissimilar materials, even without defects on the contact surfaces, creates an impedance to thermal transport that depends upon the differences in the densities and phonon propagation speeds for the two materials [51], which additionally contributes to local temperature raise. The consequence of these processes is the strong heating in small regions near individual TiB particles oriented in a certain way with respect to the applied load, which leads to local recrystallization of the matrix phases triggered adjacent to TiB particles. The situation intensifies under dynamic conditions, when the load takes place in a very short time (milliseconds), leaving no stage for the structure to recover sufficiently, all this leads to the recrystallization processes that began, which leads to the formation of nano size grains observed in Figure 21.

## 5. Conclusions

Based on the experimental data presented and their analysis, the following conclusions could be drawn:

1. The mechanical behavior of the alloy Ti64 based composites made using BEPM with different amounts of reinforcing particles: 5, 10, and 20% of TiC or 5, 10% (vol.) of TiB was analyzed taking into account the Strain Energy, phase composition, microstructure, and textures formed under dynamic and quasi static compression using strain rates in the range 10^−3^−10^3^·s^−1^.
2. All materials tested under dynamic conditions using SPB show high localization of plastic deformation in matrix alloy, mainly near reinforcement particles. Such localization was absent in materials tested under QSC.
3. The measured Strain Energy and Maximal Strain Rates demonstrate the highest values for the cast and wrought alloy Ti64, followed by Ti64BEPM + 5TiC and Ti64BEPM + 10TiB, and then Ti64BEPM free of reinforcing particles.
4. At high-strain SHPB tests, Ti64BEPM + XTiC composites have higher strength then a conventional cast and wrought Ti64 alloy if the content of TiC particles does not exceed 5% (vol.) and the strain rate does not exceed 1800 s^−1^. SE for Ti64BEPM + 5TiC and Ti64BEPM + 10TiC gradually increases with the strain rates reaching a maximum at 1800 s^−1^ and 1640 s^−1^, respectively, and then drops sharply as a result of brittle fracture. Ti64BEPM + 20TiC fractures at progressively lower strain rates, less than 1470 s^−1^, and due to a significant decrease in strength and ductility, its SE level is lower than that of Ti64BEPM + 5TiC. At lower strain rates, SE of Ti64BEPM + XTiB composites is lower compared to Ti64BEPM + XTiC equally reinforced, but due to higher plasticity of the composites Ti64BEPM + XTiB they exceed Ti64BEPM + XTiC in SE at strain rates above 2200 s^−1^.
5. For Ti64BEPM alloy that does not contain reinforcing particles, the deformation localization mechanism changes from macro to micro level when the strain rate increases above 1800–2000 s^−1^. The localization of the macro level is characterized by plastic flow affects multiple grains forming the adiabatic shear bands. The plastic flow at the micro level is limited to separate α-phase lamellae. In Ti64BEPM +XTiC, the presence of particles gives emphasis to the localization of plastic deformation at the micro level. This is due to appearance of interfacial boundaries TiC/α and TiC/β, in addition to the interfacial boundaries α/β available in Ti64BEPM and the cast and wrought Ti64. With a higher content of TiC (20 vol.%), carbide particles became the weakest microstructure elements, which led to the brittleness of MMC and its fracture at relatively low rates (less than 1500 s^−1^) due to the simultaneous crushing of carbide particles and the formation of cracks inside the matrix.
6. The negative effect of TiC is related to the high brittleness of particles or their conglomerates, which can cause the MMC to fracture under compression with relatively low strain rates. Stress concentration is formed at the interface between the carbide fragments and the matrix. Stress concentration effect under compression is less pronounced for MMC with TiB particles, which leads to an improvement in mechanical behavior of MMC composites at a higher deformation rate.
7. Localization of plastic deformation at a high strain-rates compression of MMCs with TiB, causes intense local overheating at the interface between the alloy matrix and the particles of TiB. As a result, rapid dynamic recrystallization of matrix phases can occur locally to form grains with a size of not more than 10 nm.
8. The damping ability of materials, which is analogous to the absorption of strain energy, was measured by the analysis of resonance-frequency-damping capacity. It shows that MMCs containing TiC or TiB particles absorb sound waves better in comparison with Ti64BEPM alloy free from particles. It was explained by the presence of interphases of TiC(TiB)/α, and TiC(TiB)/β in MMCs in addition to α/β interphases in the matrix alloy. The extra interfaces additionally inhibit the dislocation motion.

## Figures and Tables

**Table 1:** Chemical and phase composition of studied materials.

##	Alloying Elements and Impurities, wt.%					TiC, or TiB, vol.%	Ti	Residual Porosity, %	Phase Composition
	Al	V	Fe	O	N				
Ti64BEPM									
1	5.94	4.06	0.16	0.19	0.007	-	Balance	1.5	α + β
Ti64BEPM + 5TiC									
2	6.02	3.9	0.14	0.2	0.009	5	÷	2.1	α + β + TiC
Ti64BEPM + 10TiC									
3	6.0	4.02	0.11	0.21	0.008	10	÷	2.4	α + β + TiC
Ti64BEPM + 20TiC									
4	6.04	4.07	0.17	0.18	0.007	20	÷	2.8	α + β + TiC
Ti64BEPM + 5TiB									
5	6.03	4.04	0.12	0.22	0.008	5	÷	3.1	α + β + TiB
Ti64BEPM + 10TiB									
6	6.02	4.01	0.13	0.21	0.009	10	÷	4.0	α + β + TiB

**Table 2:** Mechanical Properties of the Obtained Materials.

##	Tensile Properties (QST) (Rate 8·10^−4^ s^−1^)				3-Point Flexure		Young Module, GPa	Damping	Sound Frequency, Hz	Vickers Hardness, HV
	YS, MPa	UTS, MPa	El. %	RA, %	Strength, MPa	Strain, %				
Ti64BEPM										
1	932	1033	7.6	21.2	2088	16.3	123	0.000251	12,022	339
Ti64BEPM + 5TiC										
2	708	708	≤0.1	-	1680	4.2	135.2	0.000266	13,551	380
Ti64BEPM + 10TiC										
3	620	620	≤0.1	-	1350	≤0.1	137	0.000309	14,029	403
Ti64BEPM + 20TiC										
4	567	567	≤0.1	-	180	≤0.1	140.4	0.000219	14,649	425
Ti64BEPM + 5TiB										
5	844	844	≤0.1	-	980	≤0.1	134.5	0.000284	13,624	362
Ti64BEPM + 10TiB										
6	522	552	≤0.1	-	645	≤0.1	138.2	0.000322	14,219	388

**Table 3:** Microanalysis EDS data of the areas within α-lamellae containing martensitic needles of two different dispersions.

Location (in Figure 11)	Chemical Composition, wt.%				
	Al	V	Fe	Ti	Sum of Impurities
1 (11g)	6.2	4.2	1.2	Balance	<0.4
2 (11h)	4.8	11.8	1.8	Balance	<0.4

**Table 4:** Local chemical analysis of MMCs’ in spots shown in Figure 22.

##	Spot #	Phase	Composition, %							
			Ti		Al		V		C/B	
			Atomic	Weight	Atomic	Weight	Atomic	Weight	Atomic	Weight
Ti64BEPM + 10TiC (Figure 22a)										
MMC General Composition			66.18	80.63	10.68	7.43	4.62	5.98	17.51	5.35
1	1	TiC	63.89	87.02	0.07	0.05	-	-	35.91	12.27
2	2	TiC	75.63	91.97	0.68	0.47	-	-	23.61	7.20
3	3	TiC	71.80	90.56	0.36	0.26	-	-	27.76	8.78
4	4	TiC	65.42	87.43	0.11	0.09	-	-	34.27	11.49
5	5	TiC	76.81	92.84	0.16	0.11	-	-	23.01	6.98
6	6	α + β	82.17	88.94	9.73	5.94	3.32	3.82	4.79	1.30
7	7	α + β	80.89	87.15	9.08	5.53	5.24	6.02	4.79	1.30
Ti64BEPM + 10TiB (Figure 22b)										
MMC General Composition			63.21	82.32	11.68	8.03	5.15	6.24	19.17	6.01
8	1	TiB	63.01	87.38	0.10	0.08	0.85	1.26	36.04	11.28
9	2	TiB	63.80	87.22	0.78	0.60	0.09	1.59	34.32	10.59
10	3	TiB	58.50	83.99	0.80	0.65	1.80	2.75	38.89	12.61
11	4	α + β	76.65	83.14	5.44	3.33	8.79	10.15	-	-
12	5	TiAl (?)	82.58	92.12	9.14	5.74	0.02	0.03	-	-
13	6	α + β	77.66	85.88	6.09	7.16	5.67	3.53	-	-

## Data Availability

The data presented in this study are available on request from the corresponding author. The data are not publicly available because of the potential for the future invention disclosure.

## List of Acronyms

**Acronym**	**Term**
Ti64	Commercial titanium alloy containing 6 (wt.)% Al and 4% V
Ti64GL	Ti-6Al-4V alloy with globular microstructure
BEPM	Blended Elemental Powder Metallurgy
Ti64BEPM	Titanium based alloy Ti-6(wt.)%Al-4V produced using BEPM
MMC	Metal Matrix Composite
Ti64BEPM + (X)TiC	MMC on the base of Ti-6Al-4V alloy containing X(vol.%) of Titanium Carbide (TiC) produced using BEPM
Ti64BEPM + (X)TiB	MMC on the base of Ti-6Al-4V alloy containing X(vol.%) of Titanium Boride (TiB) produced using BEPM
SE	Strain Energy
QSC	Quasi-Static compression
QST	Quasi-Static tension
SHPB	Split Hopkinson Pressure Bar
SEM	Scanning Electron Microscopy
SE	Secondary Electrons
BSE	Back Scattered Electrons
EBSD	Electron Backscatter Diffraction
ASB	Adiabatic Shear Band
GAM	Grain Average Misorientation
IPFz	Inverted Pole Figure in “z” axis
TKD	Transmission Kikuchi Diffraction
$\dot{\varepsilon }$ _max_	Maximal Strain Rate for which specimens were not fractured during the SHPB tests
$\dot{\varepsilon }$ _Cr_	Critical Strain Rate for which specimens became fractured during SHPB tests first time
*ε*_upper_	Maximum strain at fracture, or for the non-cracked specimens, equal to strain at the moment of the specimen unloading
*SE_max_*	Maximal Strain Energy value obtained on SHPB impact tests at Maximal Strain Rate
YS	Yield Strength (on Tension)
UTS	Ultimate Tensile Strength (on Tension)
El	Relative elongation (on Tension)
RA	Reduction in Area (on Tension)
